# Supplementary material for: Cell biological analysis reveals an essential role for Pfcerli2 in erythrocyte invasion by malaria parasites
Source: Commun Biol. 2022 Feb 9;5:121. doi: 10.1038/s42003-022-03020-9 (PMC8828742; doi:10.1038/s42003-022-03020-9)
Supplement: Supplementary file 2 — Supplementary Information [file 42003_2022_3020_MOESM2_ESM.pdf]

SUPPLEMENTARY INFORMATION

Cell biological analysis reveals an essential role for *Pfcerli2* in erythrocyte invasion by malaria parasites.

Benjamin Liffner[¶], Juan Miguel Balbin[¶], Gerald J. Shami, Ghizal Siddiqui, Jan Strauss, Sonja Frölich, Gary K. Heinemann, Ella May Edwards, Arne Alder, Jan Stephan Wichers, Darren J. Creek, Leann Tilley, Matthew W. A. Dixon, Tim-Wolf Gilberger Danny W. Wilson[*]

* Corresponding author

E-mail: danny.wilson@adelaide.edu.au

[¶]These authors contributed equally to this work.

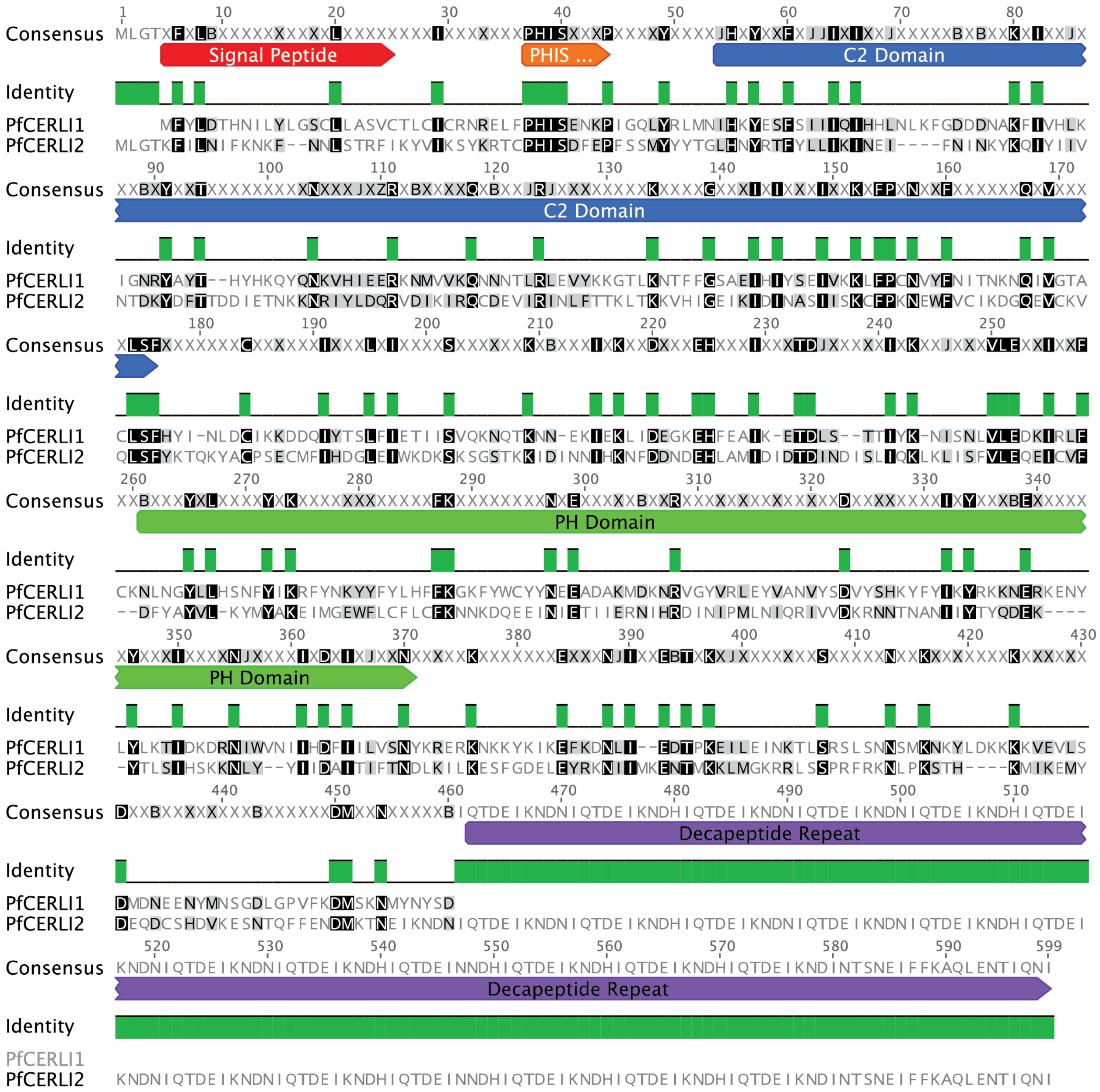

Supplementary Figure 1: Alignment of PfCERLI1 and PfCERLI2.

Geneious global alignment with free-end gaps between the amino acid sequences of PfCERLI1 (Pf3D7_0210600) and PfCERLI2 (Pf3D7_0405200). The signal peptide is predicted only for PfCERLI1 and is represented here as it is currently annotated on PlasmoDB. Removal of this signal peptide region does not affect localisation of PfCERLI1[1]*. The PH domain is predicted only for PfCERLI1, and not PfCERLI2.*

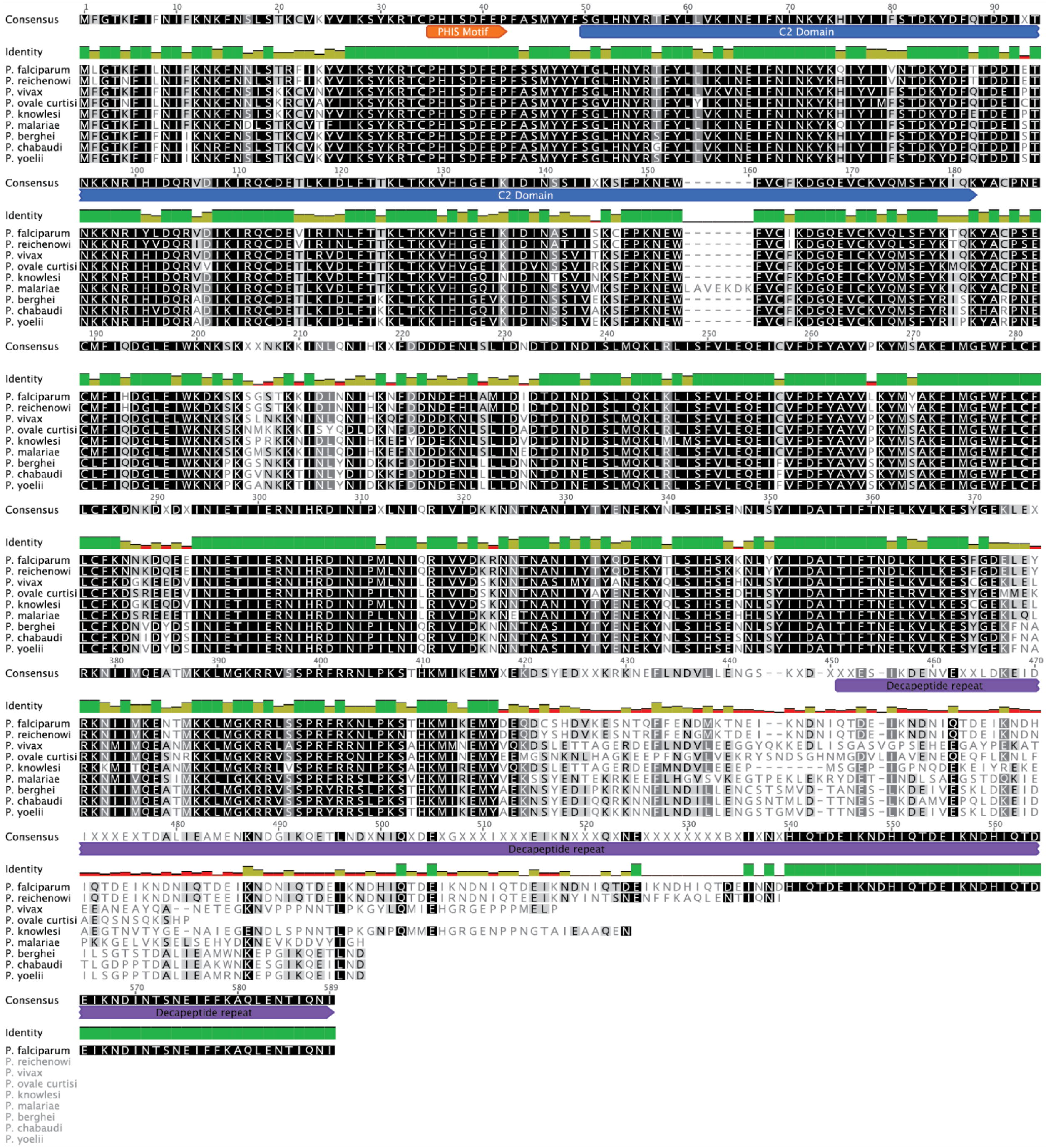

Supplementary Figure 2: Alignment of PfCERLI2 and its *Plasmodium* homologues.

Geneious global alignment with free-end gaps between the amino acid sequences of PfCERLI2 (Pf3D7_0405200) and its homologues in P. reichenowi (PRCDC_0402800), P. vivax (PVP01_0304600), P. ovale curtisi (PocGH01_030212700), P. knowlesi (PKNH_0303300) , P. malariae (PmUG01_03015300), P. berghei (PBANKA_1002900), P. chabaudi (PCHAS_1003800) , and P. yoelii (PY17X_1004300).

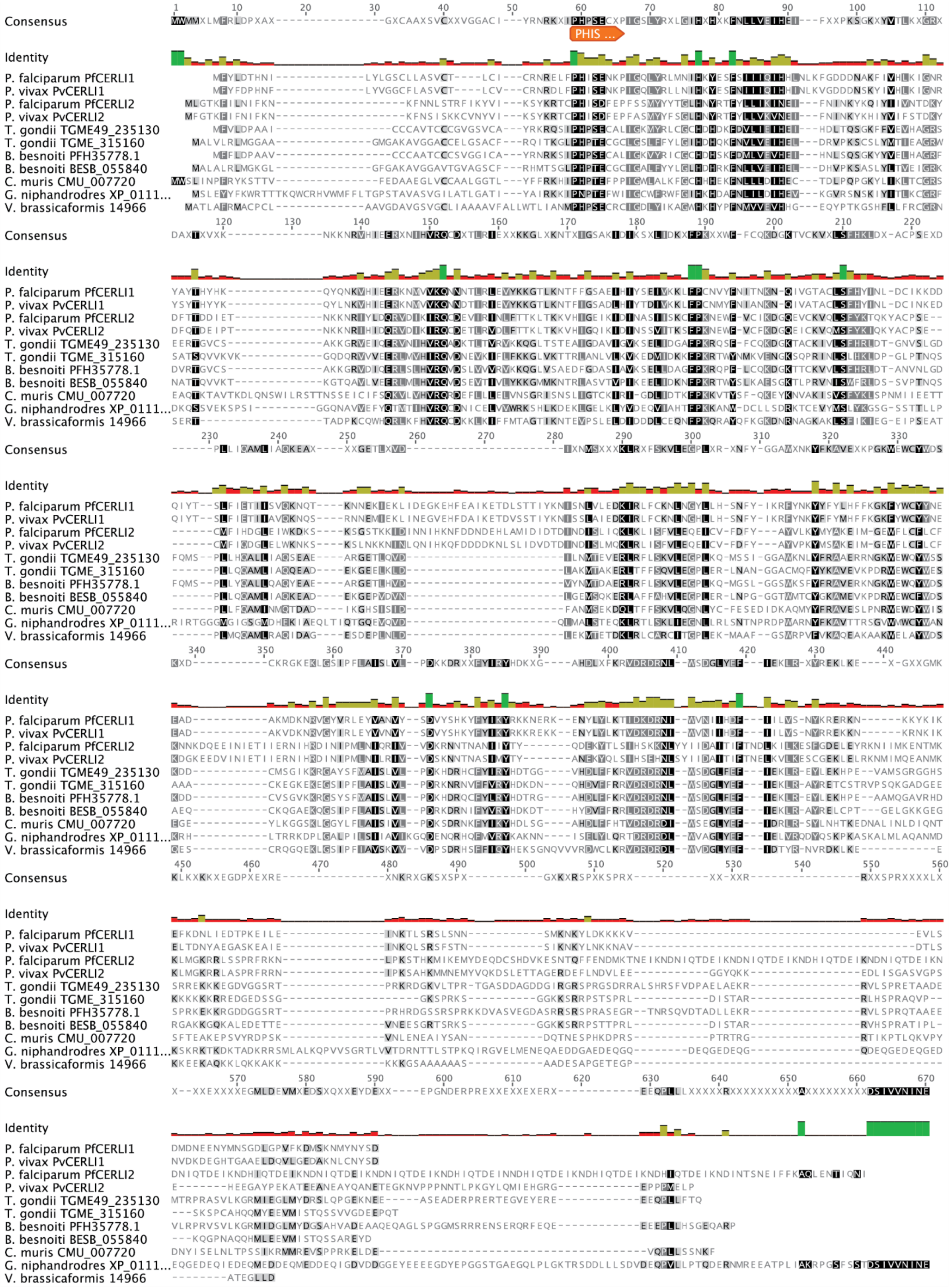
Consensus
PHIS ...
Identity
P. falciparum PfCERLI1
P. vivax PvCERLI1
P. falciparum PfCERLI2
P. vivax PvCERLI2
T. gondii TGME49_235130
T. gondii TGME_315160
B. besnoiti PFH35778.1
B. besnoiti BESB_055840
C. muris CMU_007720
G. niphandrodes XP_0111...
V. brassicaformis 14966

Supplementary Figure 3: Alignment of PfCERLI1, PfCERLI2, and their apicomplexan homologues.

Geneious global alignment with free-end gaps between the amino acid sequences of PfCERLI1 (Pf3D7_0210600), PfCERLI2 (Pf3D7_0405200) and their homologues in P. vivax (PVP01_0414300 & PVP01_0304600), Toxoplasma gondii (TGME49_235130 & TGME49_315160), Besnoitia besnoiti (PFH35778.1 & BESB_055840), Cryptosporidium muris (CMU_007720) , Gregarina niphandrodes (XP_011129839.1), and Vitrella brassicaformis (14966).

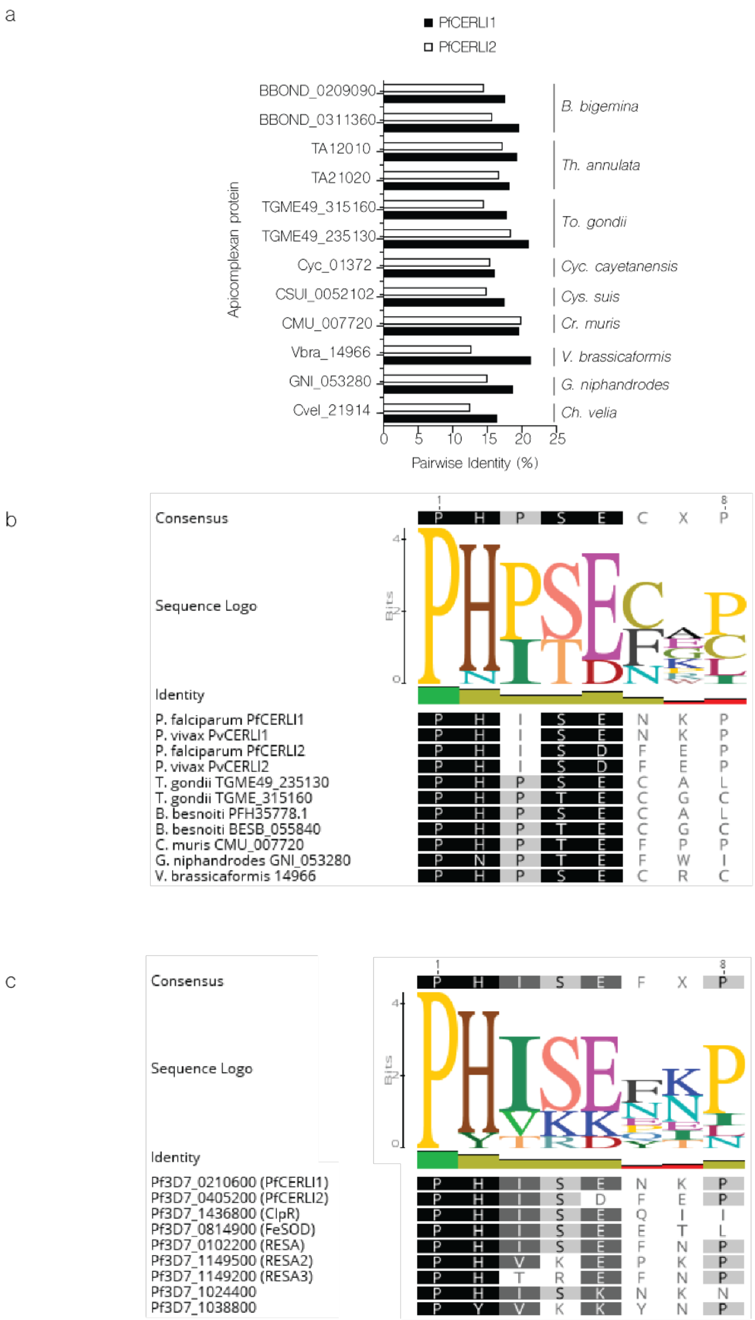
a
PfCERLI1
PfCERLI2
BBOND_0209090
BBOND_0311360
TA12010
TA21020
TGME49_315160
TGME49_235130
Cyc_01372
CSUI_0052102
CMU_007720
Vbra_14966
GNI_053280
Cvel_21914
B. bigemina
Th. annulata
To. gondii
Cyc. cayetanensis
Cys. suis
Cr. muris
V. brassicaformis
G. niphandrodes
Ch. velia
Apicomplexan protein
Pairwise Identity (%)
0 5 10 15 20 25
b
Consensus
Sequence Logo
Identity
Bits
P H P S E C X P
P. falciparum PfCERLI1
P. vivax PvCERLI1
P. falciparum PfCERLI2
P. vivax PvCERLI2
T. gondii TGME49_235130
T. gondii TGME_315160
B. besnoiti PFH35778.1
B. besnoiti BESB_055840
C. muris CMU_007720
G. niphandrodes GNI_053280
V. brassicaformis 14966
c
Consensus
Sequence Logo
Identity
Bits
P H I S E F X P
Pf3D7_0210600 (PfCERLI1)
Pf3D7_0405200 (PfCERLI2)
Pf3D7_1436800 (ClpR)
Pf3D7_0814900 (FeSOD)
Pf3D7_0102200 (RESA)
Pf3D7_1149500 (RESA2)
Pf3D7_1149200 (RESA3)
Pf3D7_1024400
Pf3D7_1038800

Supplementary Figure 4: Pairwise identity of PfCERLI1, PfCERLI2, and their homologues.

(a) *Pairwise identities, as determined by Geneious global alignment with free-end gaps, of the amino acid sequence for either PfCERLI1 or PfCERLI2 and their homologues in Babesia bigemina (BBOND_0209090 & BBOND_0311360), Theileria annulata (TA12010 & TA21020), Toxoplasma gondii (TGME49_235130 & TGME49_315160), Cyclospora cayetanensis (Cyc_01372), Cystoisospora suis (CSUI_0052102), Cryptosporidium muris (CMU_007720), Vitrella brassicaformis (Vbra_14966), Gregarina niphandrodes (GNI_053280), and Chromera velia (Cvel_21914).* ***(b)*** *Consensus sequence and conservation of the PHIS motif in PfCERLI1, PfCERLI2, and their apicomplexan homologues. (****c)*** *Consensus sequence and conservation of the PHIS motif for PHIS-containing proteins of P. falciparum.*

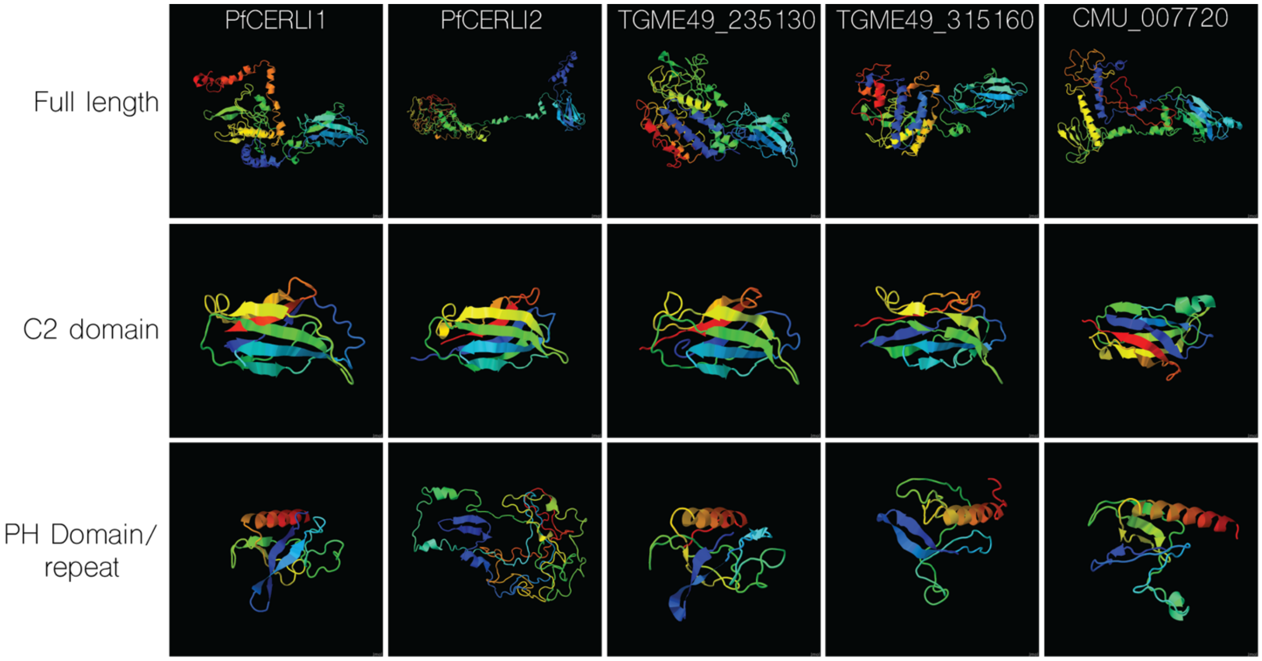

Supplementary Figure 5: Predicted protein structures of CERLI1, CERLI2 and their homologues.

Full length protein structure of PfCERLI1, PfCERLI2, their T. gondii homologues (TGME49_235130 and TGME49_315160), and their Cryptosporidium muris homologue (CMU_007720) was predicted using Phyre2. All proteins were predicted to have a C2 domain, and all proteins except PfCERLI2 were also predicted to contain a Pleckstrin homology (PH) domain.

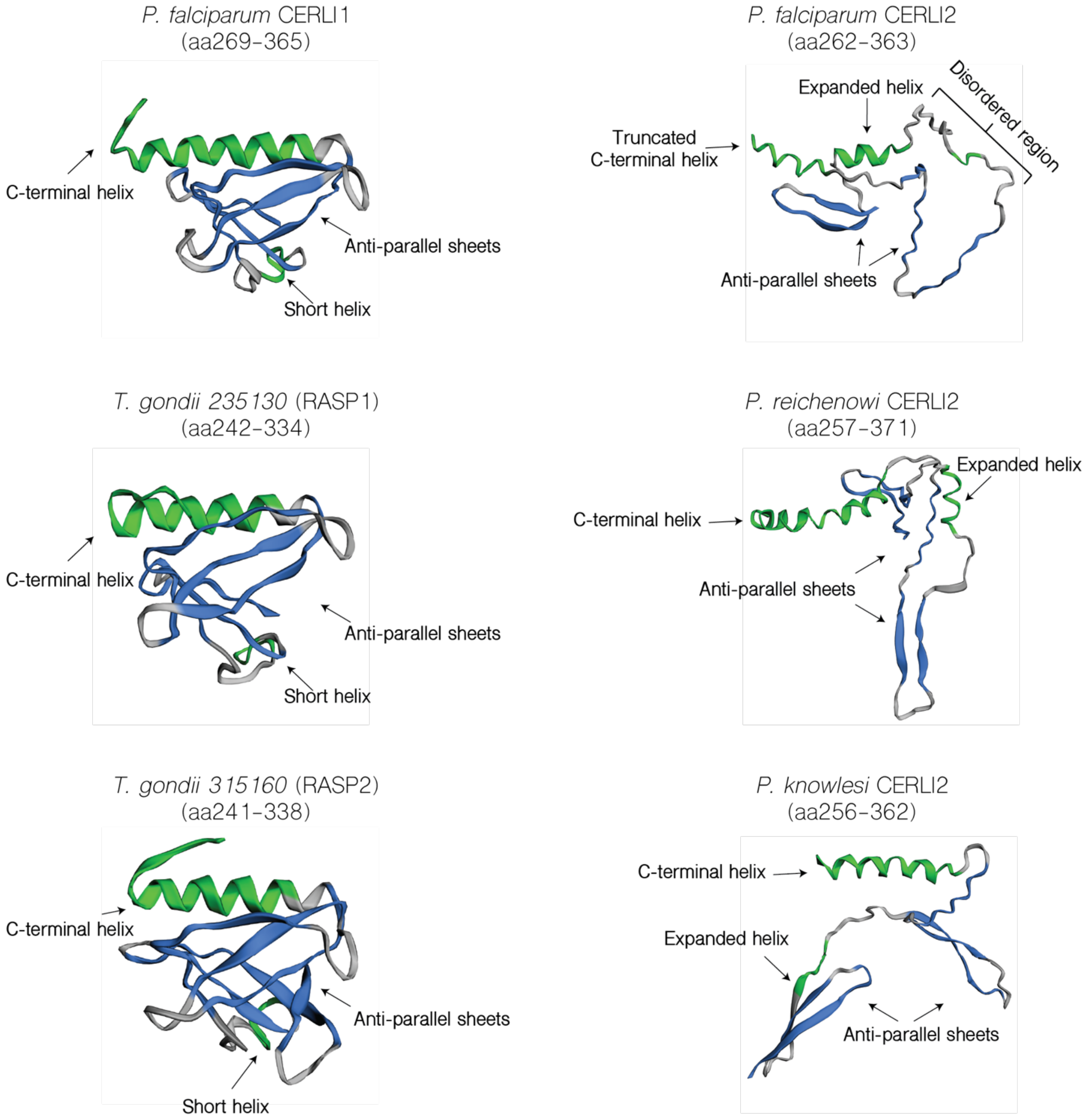

Supplementary Figure 6: Predicted structure of the PH domain region of PfCERLI2, PfCERLI1 and their homologues.

Full length protein sequences for PfCERLI1, PfCERLI2, along with both their homologues in Toxoplasma gondii, and the homologues of CERLI2 in Plasmodium reichenowi and Plasmodium knowlesi, were predicted using Phyre2. The PH domain region of those structures was visualised using EzMol. Region of each protein depicted are shown in brackets. Green = alpha-helix, blue = beta-strand, grey = coil.

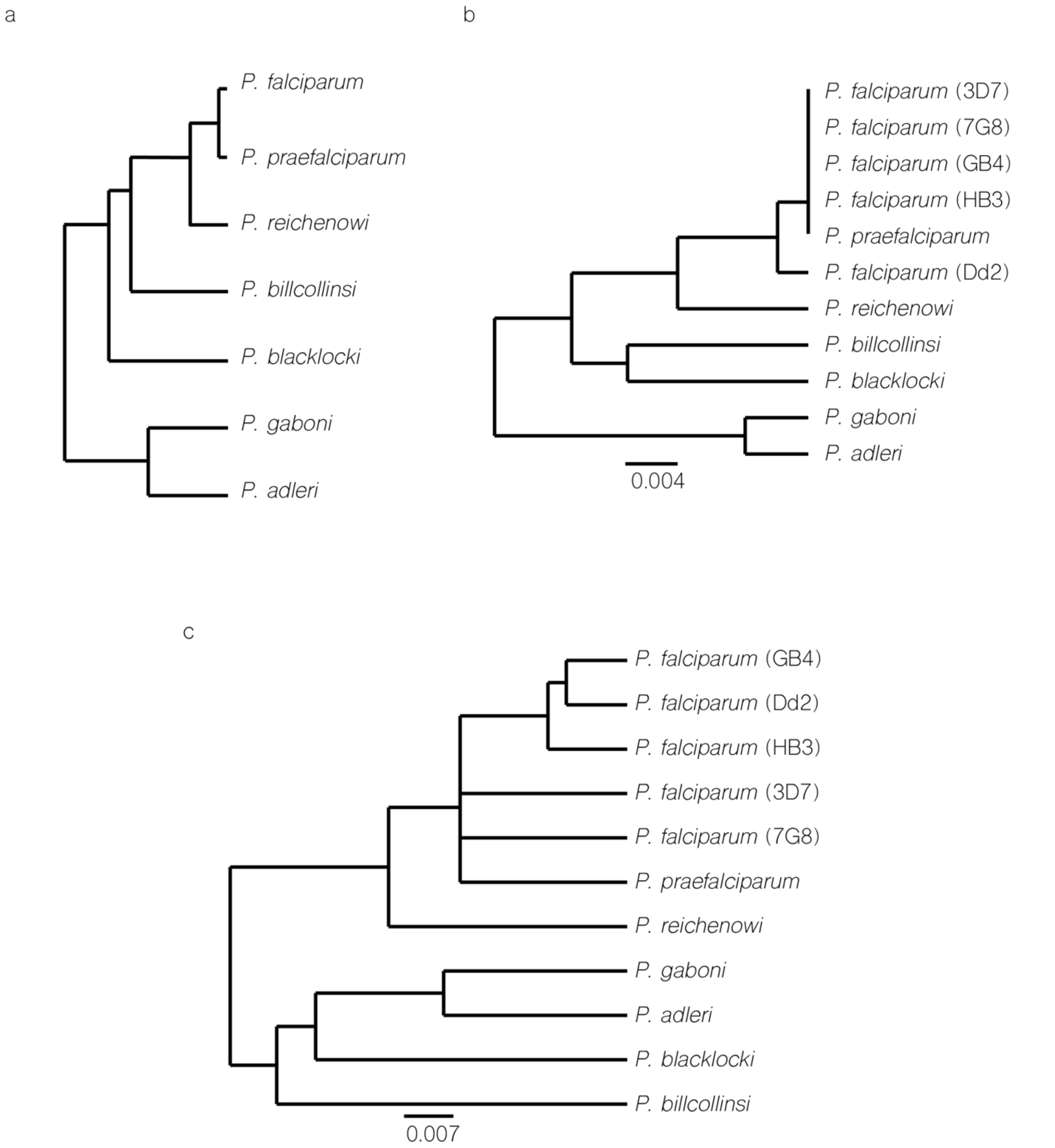

Supplementary Figure 7: The decapeptide repeat of PfCERLI2 is under differential selection in *Laverania*.

(a) *Cladogram of relationships between Laverania as determined by Otto et al., Nat. Microb. 2018.* ***(b)*** *Phylogenetic tree of PfCERLI2 and its homologues in Laverania and multiple P. falciparum isolates when the decapeptide repeat region of each protein has been removed.* ***(c)*** *Phylogenetic tree of full length PfCERLI2 and its homologues in Laverania and multiple P. falciparum. Scale bars = amino acid substitutions per site. Phylogenetic trees constructed using unweighted pair group method with arithmetic mean (UPGMA). Branch length corresponds to amino acids substitutions per site.*

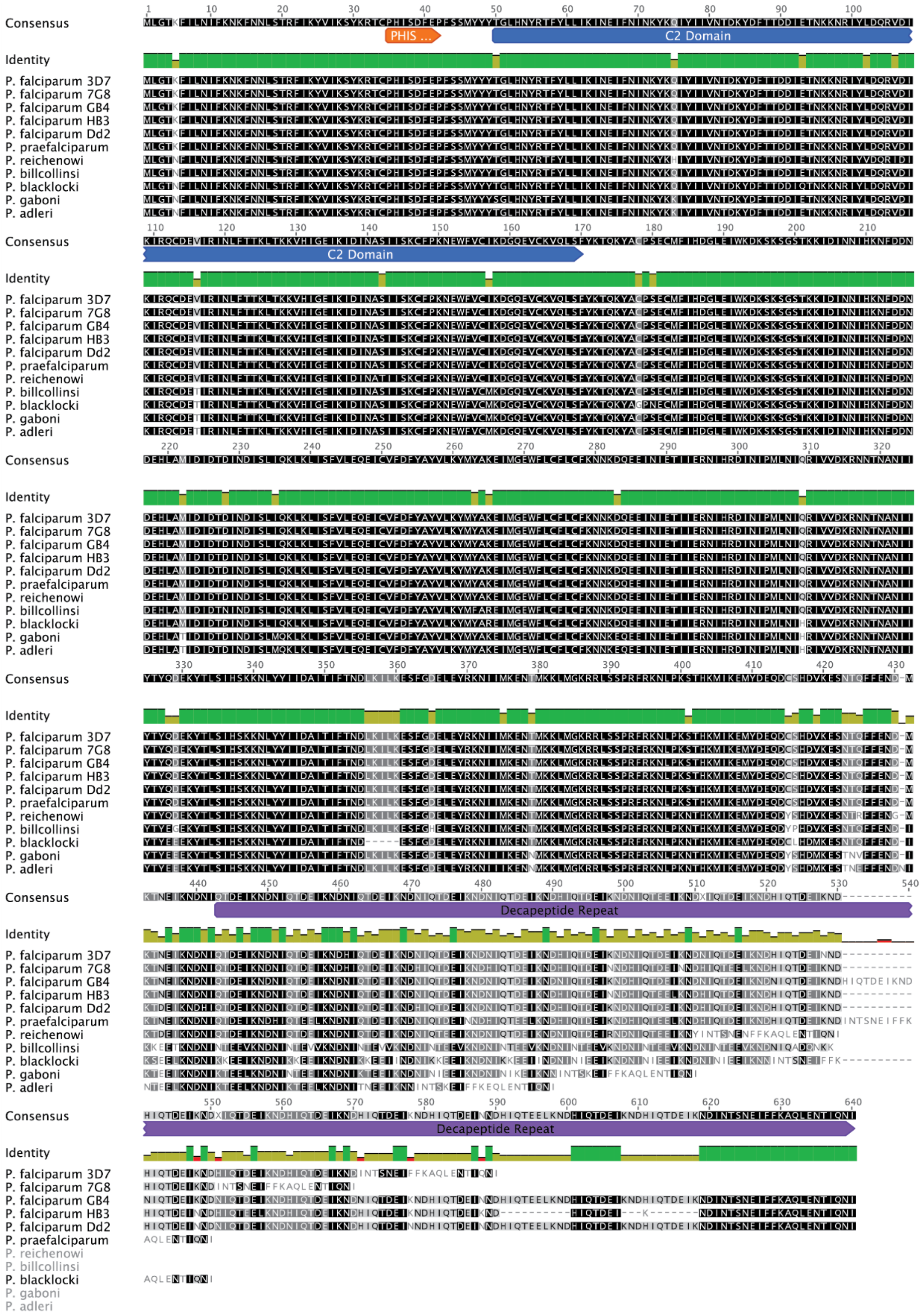

Supplementary Figure 8: Alignment of PfCERLI2 and its homologues in *Laverania*.

Geneious global alignment with free-end gaps between full length PfCERLI2 in Laverania and multiple P. falciparum isolates.

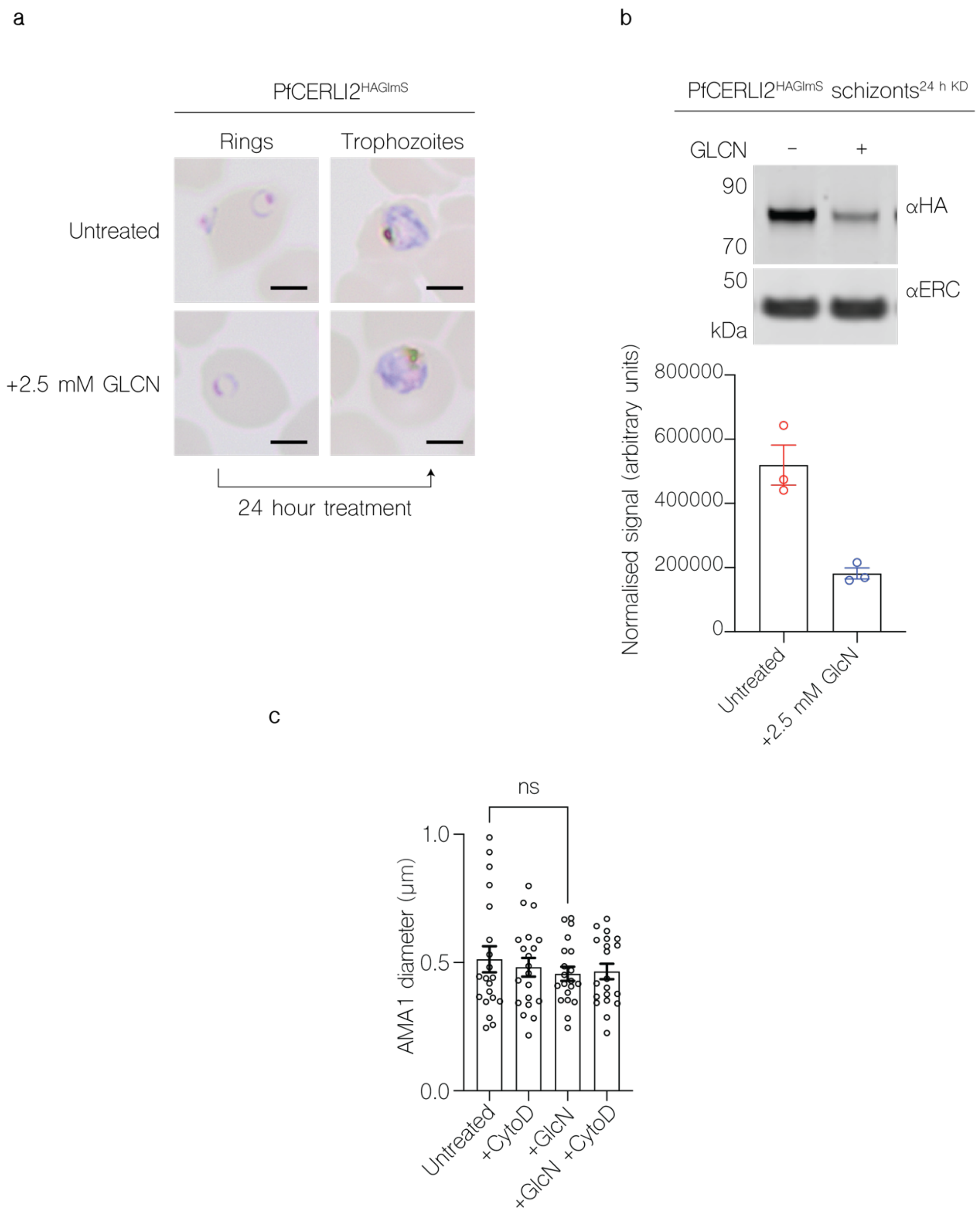
a
PfCERLI2HAGlmS
Rings
Trophozoites
Untreated
+2.5 mM GLCN
24 hour treatment
b
PfCERLI2HAGlmS schizonts24 h KD
GLCN
–
+
90
70
50
kDa
αHA
αERC
Normalised signal (arbitrary units)
800000
600000
400000
200000
0
Untreated
+2.5 mM GlcN
c
ns
AMA1 diameter (µm)
1.0
0.5
0.0
Untreated
+CytoD
+GlcN
+GlcN +CytoD

Supplementary Figure 9: Assessment of PfCERLI2^{HAGlmS} expression, growth and invasion under GLCN-inducible knockdown or cytochalasin D treatment.

(a) *PfCERLI2^{HAGlmS} ring stages (0-6 hrs) were treated with 2.5 mM GLCN or left untreated, and allowed to grow for 24 hours into trophozoites (24-30 hrs). Samples were smeared onto glass slides, blinded and imaged by light microscopy (n=5 images taken per condition, two biological replicates). Representative micrographs show no obvious defects in trophozoite morphology in response to GLCN treatment. Scale bars represent 2 μm.* ***(b)*** *PfCERLI2^{HAGlmS} trophozoites (24 hrs) were treated with 2.5 mM GLCN or left untreated, allowed to grow until schizonts had fully developed to quantify PfCERLI2 GLCN-inducible knockdown over a 24 hour period. Densitometry shows a 65% reduction in PfCERLI2 signal (n=3 biological replicates).* ***(c)*** *PfCERLI2^{HAGlmS} ring stages were treated with 2.5 mM GLCN or left untreated, allowed to mature into schizonts in the presence of E64 and purified as free merozoites. Free merozoites, with or without GLCN-induced knockdown of PfCERLI2, were permitted to invade RBCs treated with or without 1 μM cytochalasin D. This graph represents the full data set presented in Figure 3 of the manuscript without exclusions. n=20 merozoites in a single biological replicate, ns=p>0.05 by unpaired t-test. Error bars = SEM.*

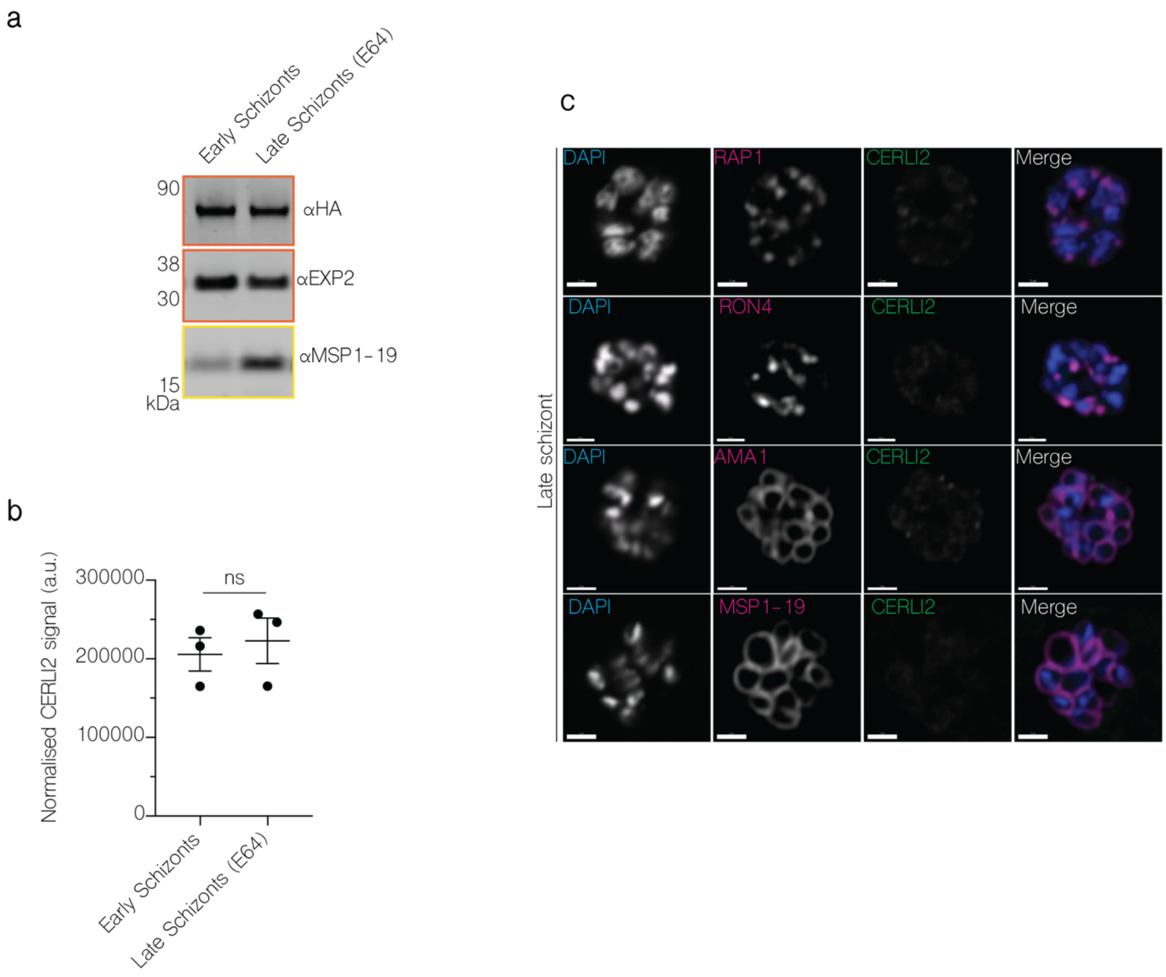

Supplementary Figure 10: Assessment of HA-tagged PfCERLI2 signal visibility.

(a) *PfCERLI2^{HAGlmS} parasite lysates were either harvested for Western blot as early schizonts (~44 hours post invasion), or matured in the presence of E64 and harvested as late schizonts (~48). Lysates were probed with either an antibody cocktail containing anti-HA (PfCERLI2), anti-EXP2 (loading control) (outlined in orange), or anti-MSP1-19 (schizont maturity control)(outlined in yellow) antibodies. Image representative of 3 biological replicates.* ***(b)*** *Quantification of PfCERLI2 signal, normalised to EXP2, for both early and late schizonts. n=3, ns=p>0.05 by unpaired t-test. Error bars = SEM.* ***(c)*** *Immunofluorescence microscopy of late PfCERLI2^{HAGlmS} schizonts stained with DAPI (nucleus) and anti-HA (PfCERLI2) antibodies, along with antibodies to either RAP1 (rhoptry bulb, RON4 (rhoptry neck), AMA1 (micronemes), or MSP1-19 (merozoite surface). Scale bar = 2 μm*

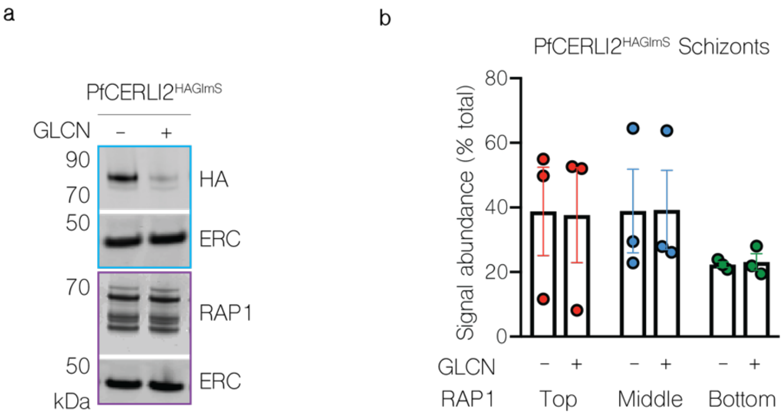

Supplementary Figure 11: PfCERLI2 knockdown does not alter RAP1 processing in C1 arrested schizonts.

(a) *PfCERLI2^{HAGlmS} parasites were either treated with 2.5 mM GLCN or left untreated, before being matured in the presence of C1 to prevent PVM rupture. Parasite lysates were then prepared and probed with a cocktail of anti-HA (PfCERLI2) and anti-ERC antibodies (outlined in blue), and anti-RAP1 (rhoptry bulb) and anti-ERC (outlined in purple) antibodies. Images representative of 3 biological replicates.* ***(b)*** *Quantification of individiual band intensities, as a percentage of the total signal, from RAP1 signals using the parasite lysates from the secretion assay. n=3 biological replicates. Error bars = SEM.*

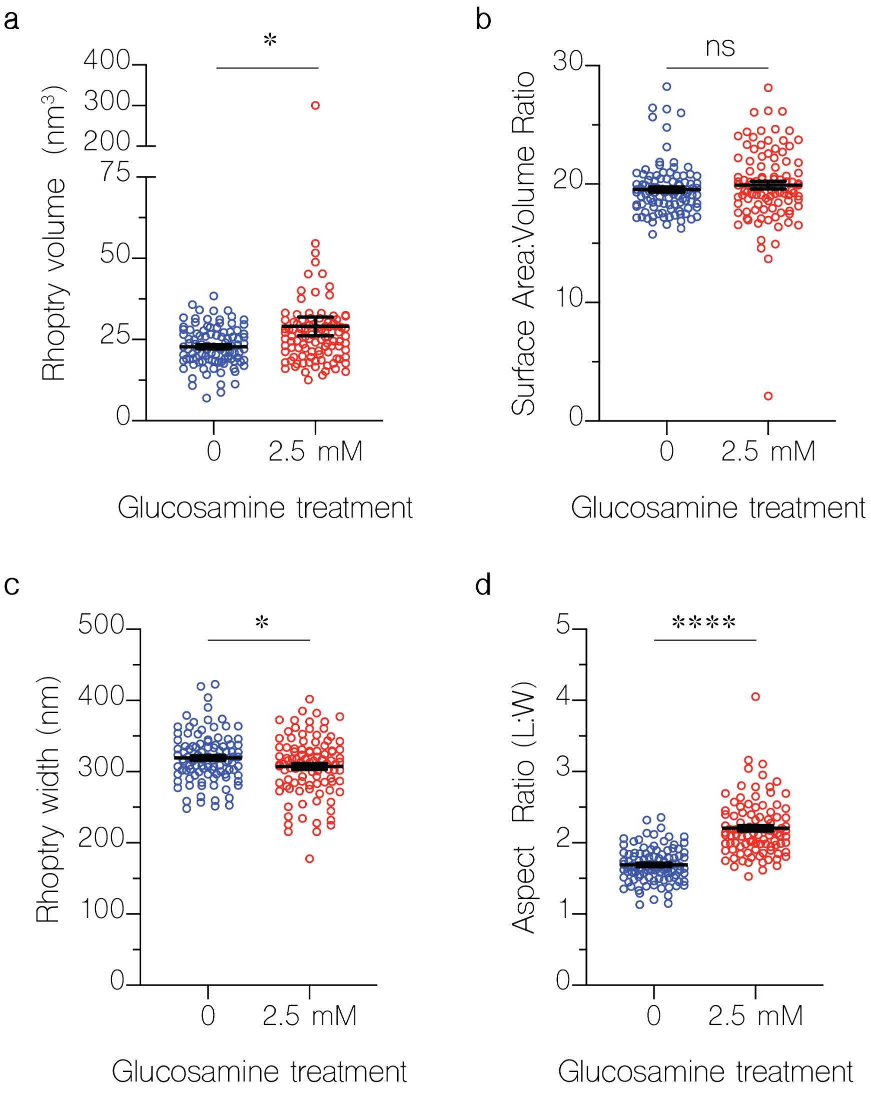

Supplementary Figure 12: PfCERLI2 knockdown grossly alters rhoptry morphology.

PfCERLI2^{HAGlmS} parasites were either treated with 2.5 mM GLCN, or left untreated, from ring-stages and arrested at schizont stages using C1. These parasites were then imaged by serial block face scanning electron microscopy (SBF-SEM) array tomography. 100 rhoptries for each treatment were segmented, with their volume ***(a)****, surface are to volume ratio* ***(b)****, width* ***(c)****, and length to width aspect ratio* ***(d)*** *calculated. Error bars = SEM. ns = $p>0.05$, *=$p<0.05$, ****=$p<0.0001$ by unpaired t-test.*

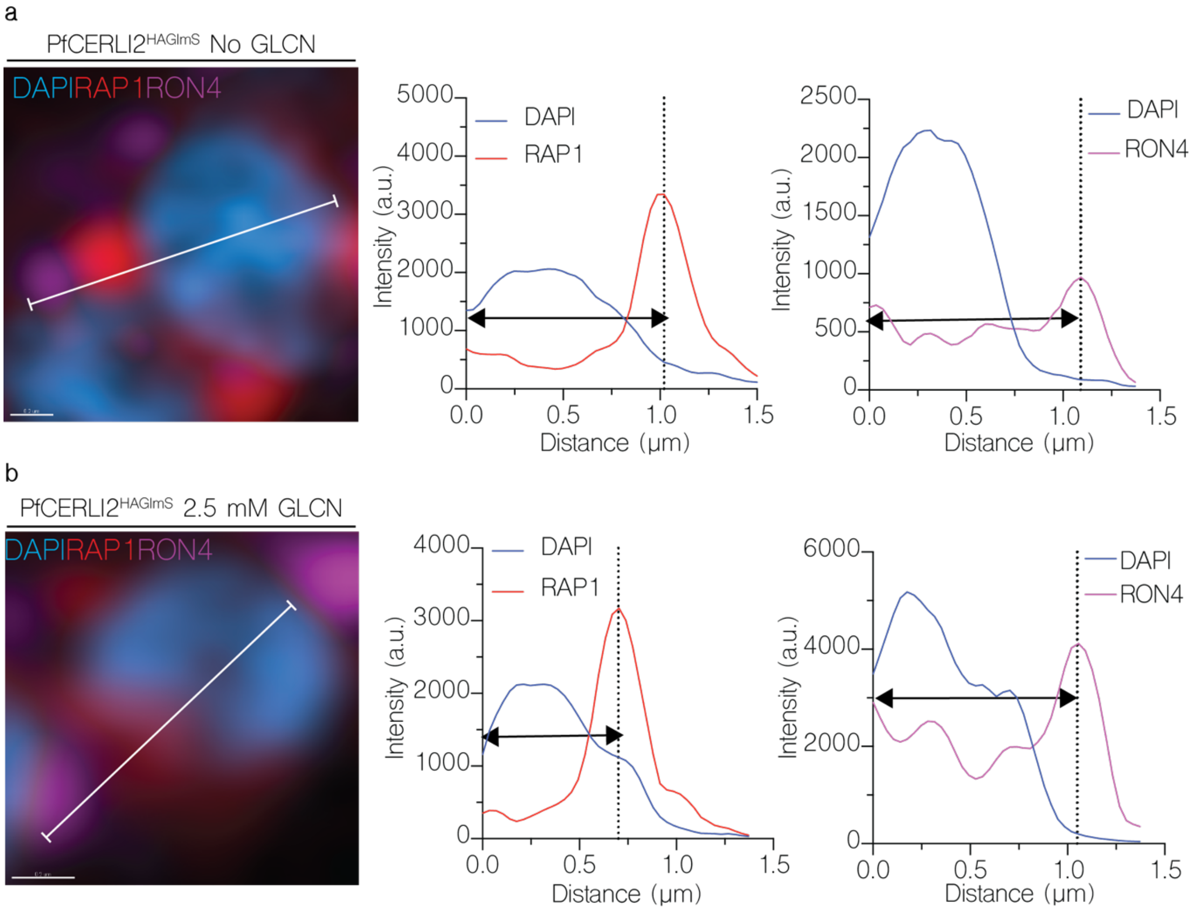

Supplementary Figure 13: Knockdown of PfCERLI2 alters rhoptry antigen positioning and distribution.

PfCERLI2^{HAGlmS} schizonts were matured in the presence of E64, stained with antibodies against RAP1 (rhoptry bulb) and RON4 (rhoptry neck), and imaged by Airyscan super-resolution microscopy. The flourescence intensity from maximum-intensity projections of RAP1 and RON4 signals were then measured from the basal end of the nucleus and plotted against merozoite length for parasites that were either left untreated ***(a)*** *or treated with 2.5 mM GLCN* ***(b)****. Dashed lines = RAP1/RON4 maximum fluorescence intensity. Scale bars represent 0.2 µm.*

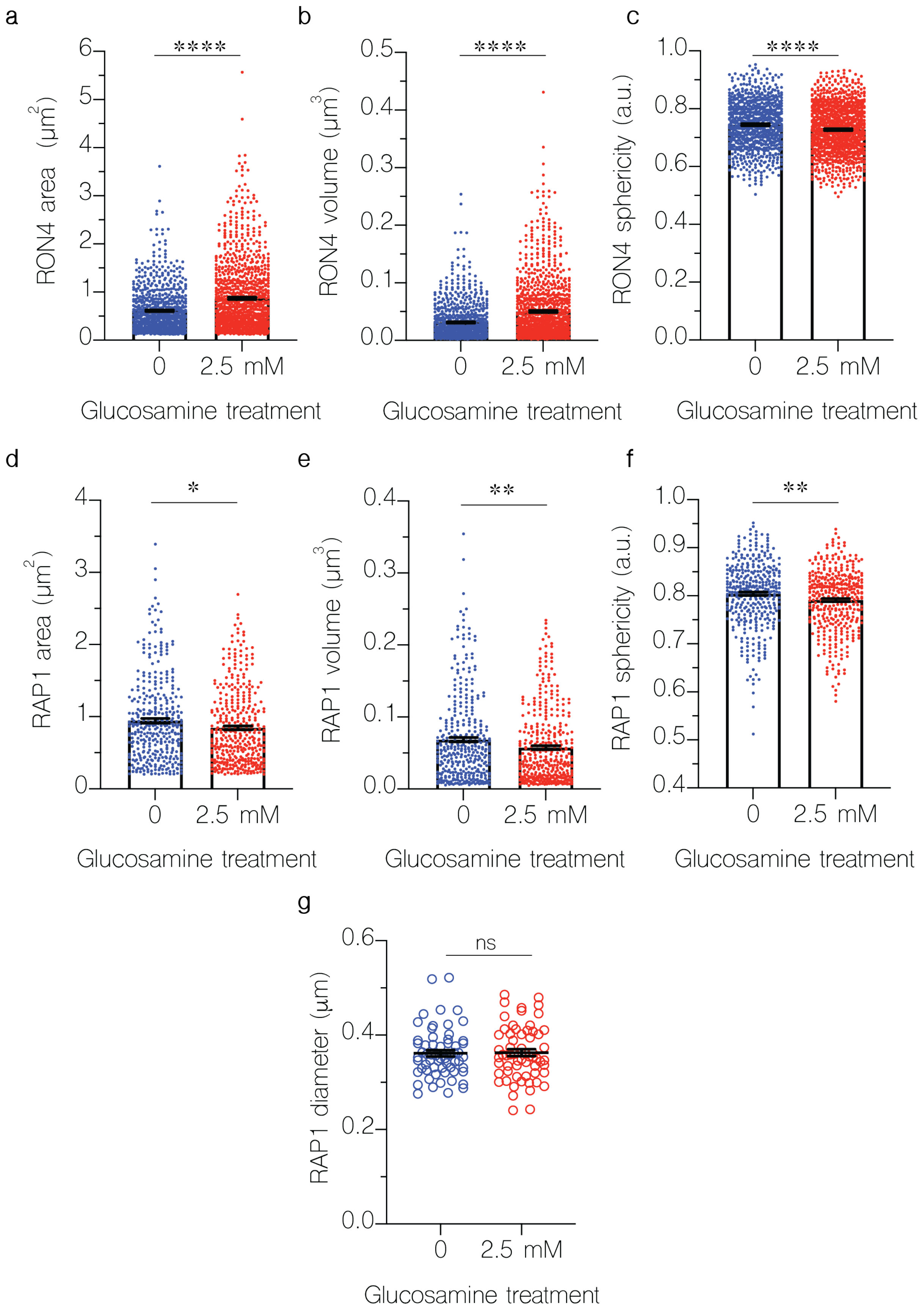
a
b
c
****
RON4 area (μm2)
RON4 volume (μm3)
RON4 sphericity (a.u.)
0
2.5 mM
Glucosamine treatment
d
e
f
*
**
RAP1 area (μm2)
RAP1 volume (μm3)
RAP1 sphericity (a.u.)
g
ns
RAP1 diameter (μm)

172

173

Supplementary Figure 14: PfCERLI2 knockdown alters the distribution of rhoptry markers.

*$PfCERLI2^{HAGlmS}$ parasites were either treated with 2.5 mM GLCN from ring-stages until schizont stages, or left untreated. Schizonts were then matured in the presence of the egress inhibitor E64. Parasites were then stained with DAPI, anti-RAP1 (rhoptry bulb), and anti-RON4 (rhoptry neck) antibodies, and analysed by Airyscan super-resolution microscopy. RON4 foci area **(a)**, volume **(b)** and sphericity **(c)**, along with RAP1 foci area **(d)**, volume **(e)**, and sphericity **(f)** were then quantified using an established automated image analysis pipeline for these markers[1]. 779 foci quantified for RON4 untreated, 1042 foci quantified for RON4 + 2.5 mM GLCN, 367 foci quantified for RAP1 untreated, 386 foci quantified for RAP1 + 2.5 mM GLCN. **(g)** Images of the same parasites were then blinded and the rhoptry bulb diameter (RAP1 signal) measured, with each datapoint representing a single rhoptry. 60 rhoptries were measured for untreated parasites, and 62 for + 2.5 mM GLCN parasites. N=3, all error bars = SEM, ns = $p>0.05$, * = $p<0.05$, ** = $p<0.01$, **** = $p<0.0001$ by unpaired t-test.*

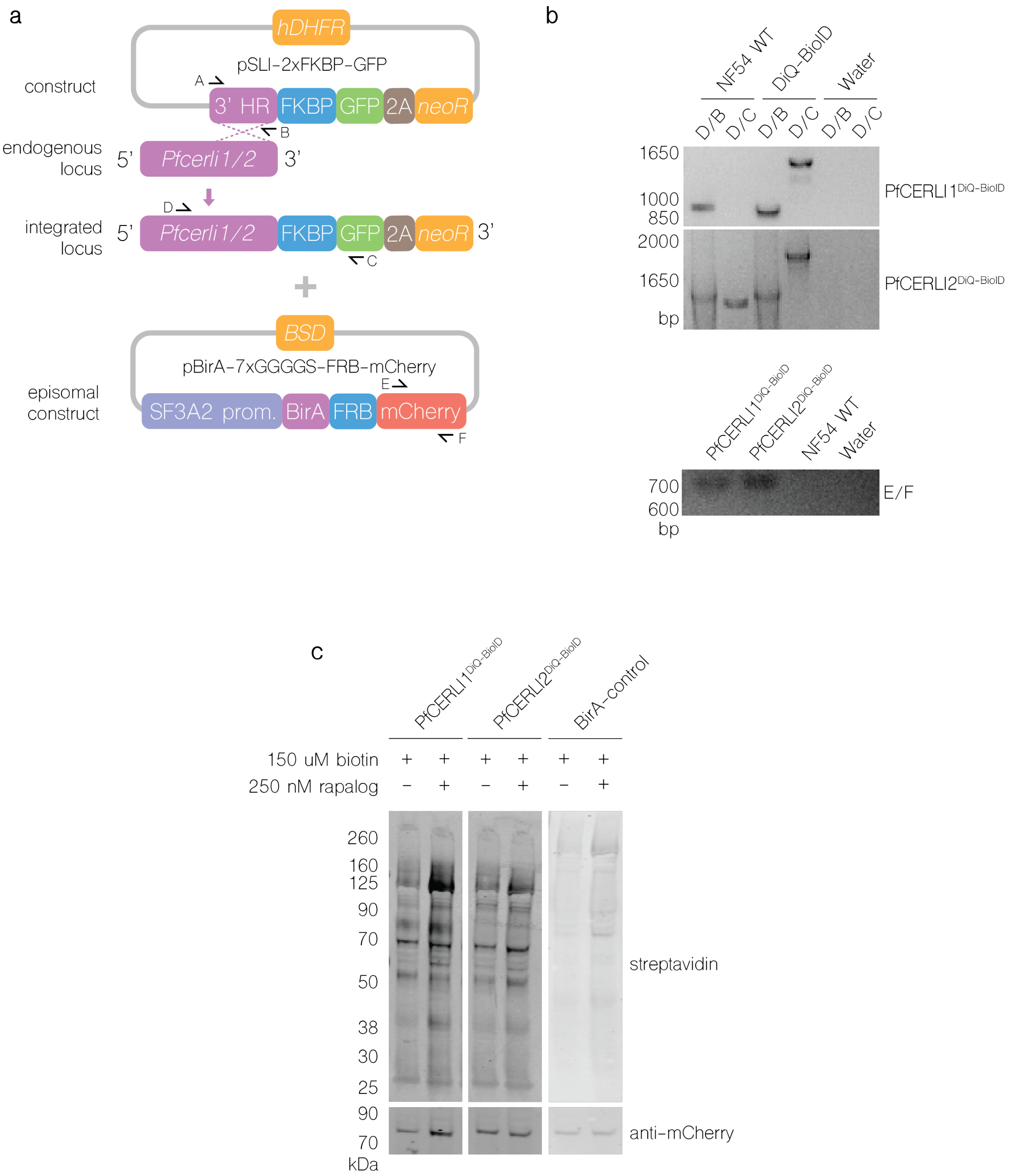
a
hDHFR
pSLI-2xFKBP-GFP
construct
A
3' HR
FKBP
GFP
2A
neoR
B
endogenous locus
5'
Pfcerli1/2
3'
D
integrated locus
5'
Pfcerli1/2
FKBP
GFP
2A
neoR
3'
C
BSD
pBirA-7xGGGGS-FRB-mCherry
E
episomal construct
SF3A2 prom.
BirA
FRB
mCherry
F
b
NF54 WT
DiQ-BioID
Water
D/B
D/C
D/B
D/C
D/B
D/C
1650
1000
850
2000
1650
bp
PfCERLI1DiQ-BioID
PfCERLI2DiQ-BioID
PfCERLI1DiQ-BioID
PfCERLI2DiQ-BioID
NF54 WT
Water
700
600
bp
E/F
c
PfCERLI1DiQ-BioID
PfCERLI2DiQ-BioID
BirA-control
150 uM biotin
250 nM rapalog
+ + + + + +
– + – + – +
260
160
125
90
70
50
38
30
25
90
70
kDa
streptavidin
anti-mCherry

Supplementary Figure 15. Gene editing strategy for generating PfCERLI1$^{\text{DiQ-BioID}}$ and PfCERLI2$^{\text{DiQ-BioID}}$ lines and validation of construct expression.

(a) PfNF54 parasites were first transfected with a selection-linked integration (SLI) construct used for endogenously integrating an FKBP-GFP tag onto the C-termini of PfCERLI1 and PfCERLI2. This was followed by transfection with an episomal construct expressing an FRB-mCherry tagged BirA biotinylator. (b) Confirmation of SLI construct integration and episomal maintenance of the biotinylator plasmid by PCR. Primer positions are denoted by arrows in (a). (c) PfCERLI1$^{\text{DiQ-BioID}}$, *PfCERLI2*$^{\text{DiQ-BioID}}$ *and control lines were treated with biotin with or without rapalog and analysed by Western blot. Blot was probed with a streptavidin-conjugated IRDye800 to visualise biotinylated proteins, and an anti-mCherry antibody to visualise the BirA-FRB-mCherry fusion protein at approximately ~70 kDa.*

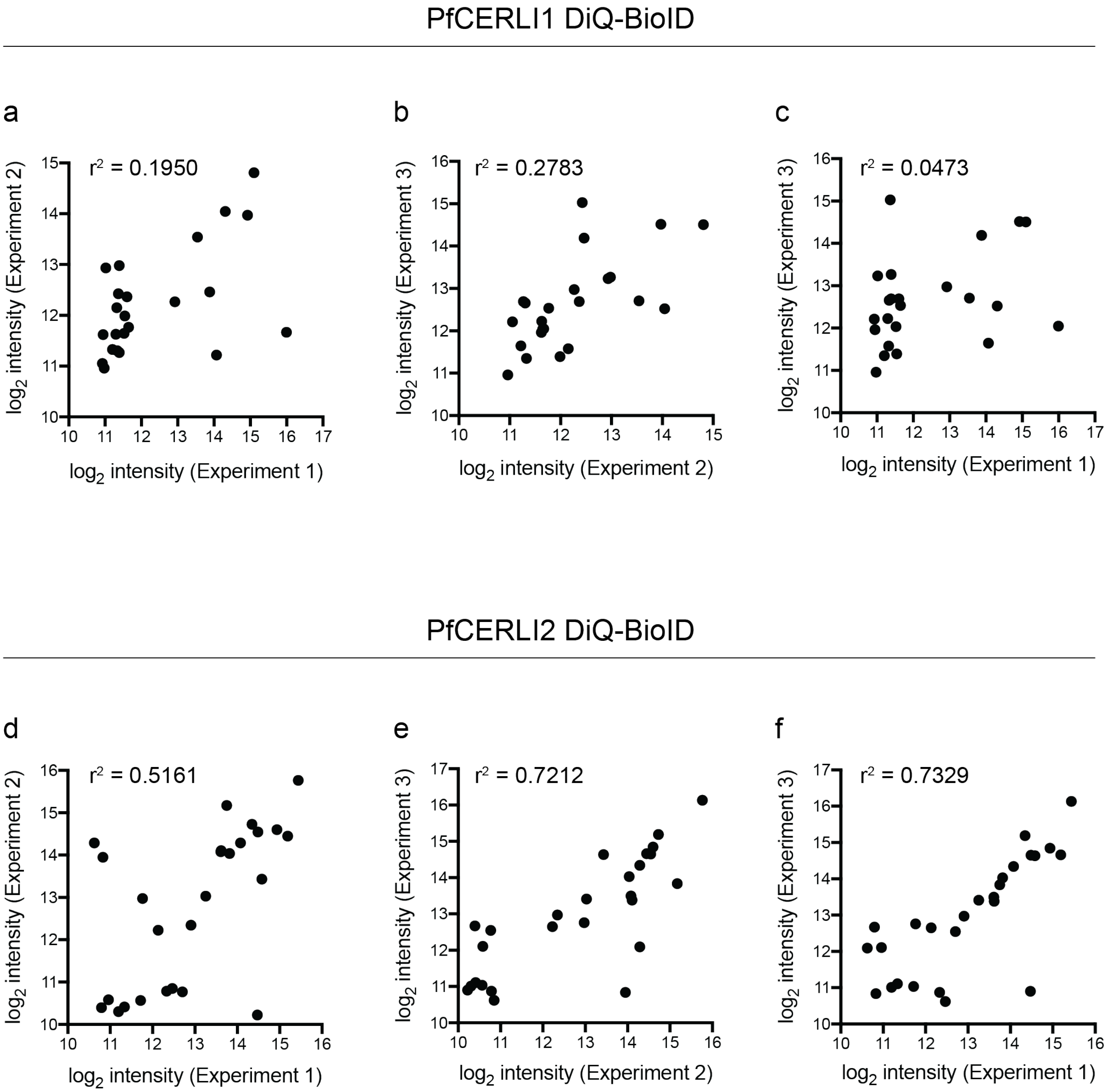

Supplementary Figure 16. Assessment of experimental reproducibility by linear correlation for dimerization inducible quantitative biotin identification (DiQ-BioID) intensity (abundance) values of:

(a-c) PfCERLI1 and (d-f) PfCERLI2 for proteins identified with at least 2 peptides and 10% or greater library coverage, present in samples treated with both biotin and rapalog. Data represents all three biological replicates for each protein.

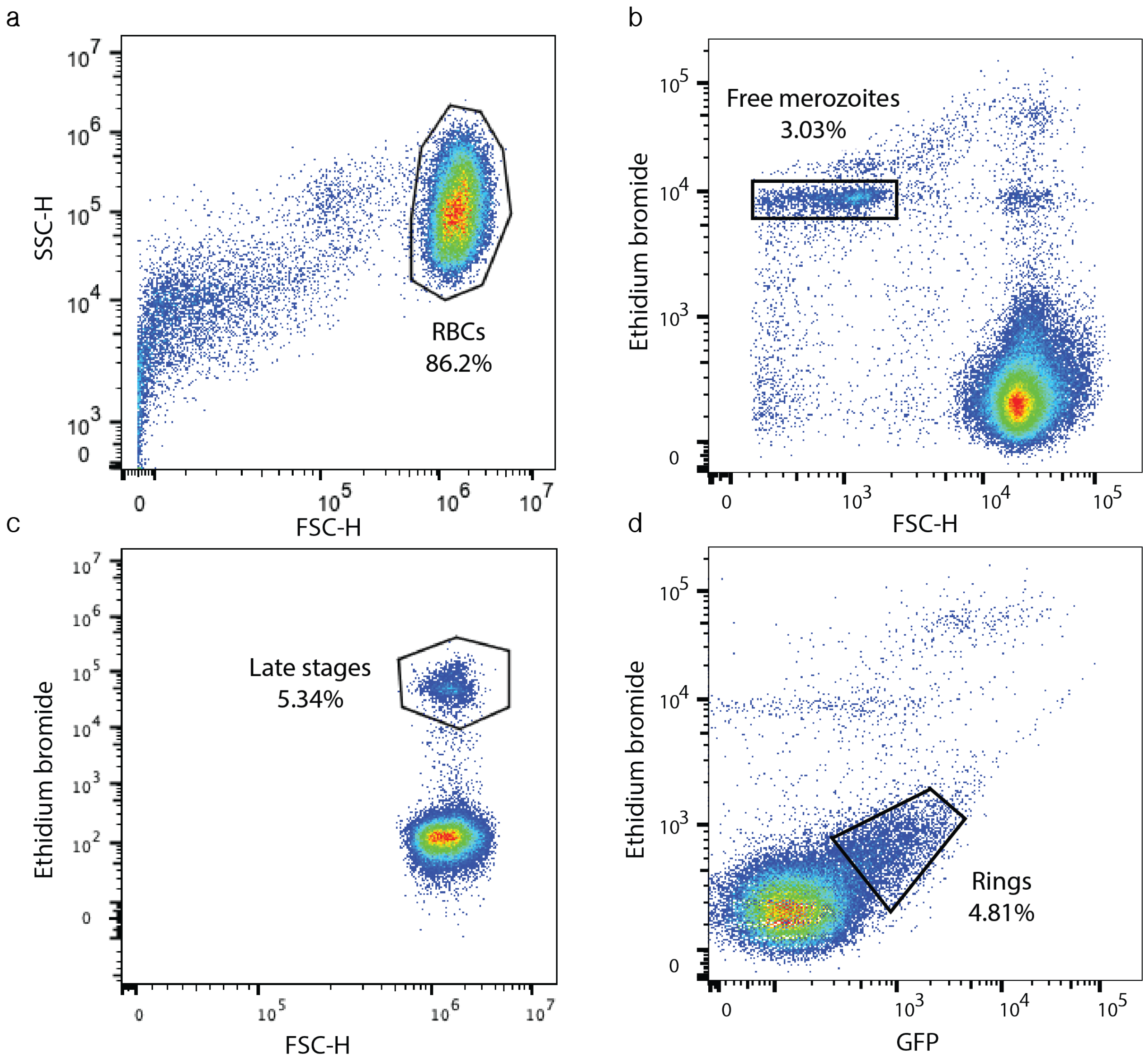

Supplementary Figure 17. Representative flow plots showing gating strategies for flow cytometry experiments performed in this study.

Plots were exported from FlowJo Version 10. (a), gating for red blood cells (RBCS) (b) free merozoites (c), late-stage parasites, including trophozoites and schizonts (d), GFP-fluorescent ring stage parasites.

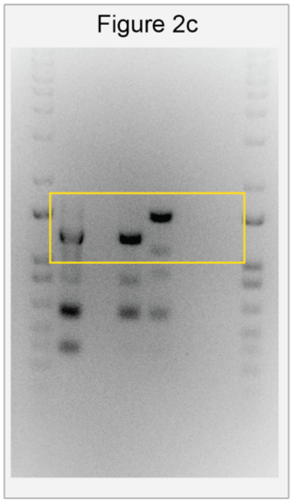
Figure 2c

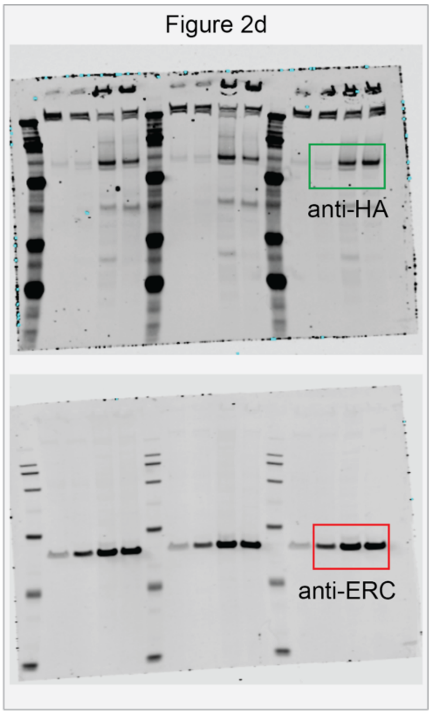
Figure 2d
anti-HA
anti-ERC

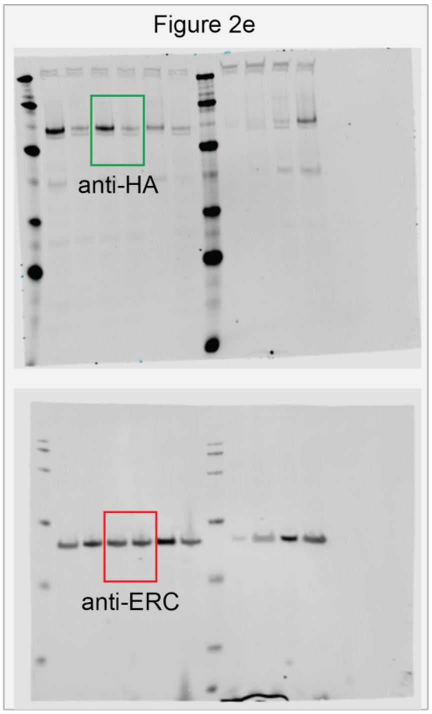
Figure 2e
anti-HA
anti-ERC

Supplementary figure 18. Full agarose gels and western blots used in Figure 2.

Corresponding figures and antibodies used are annotated. Bands that were cropped in Figure 2 have been boxed according to the colour theme: yellow (DNA gel), green (detected in the 800 nm channel), red (detected in the 700 nm channel).

Figure 5a

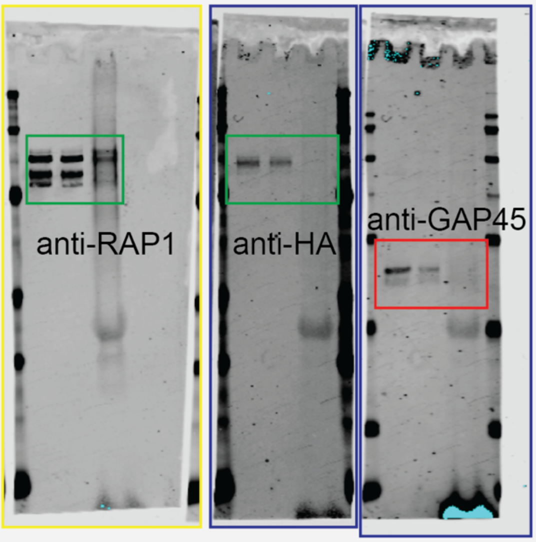

Figure 5b

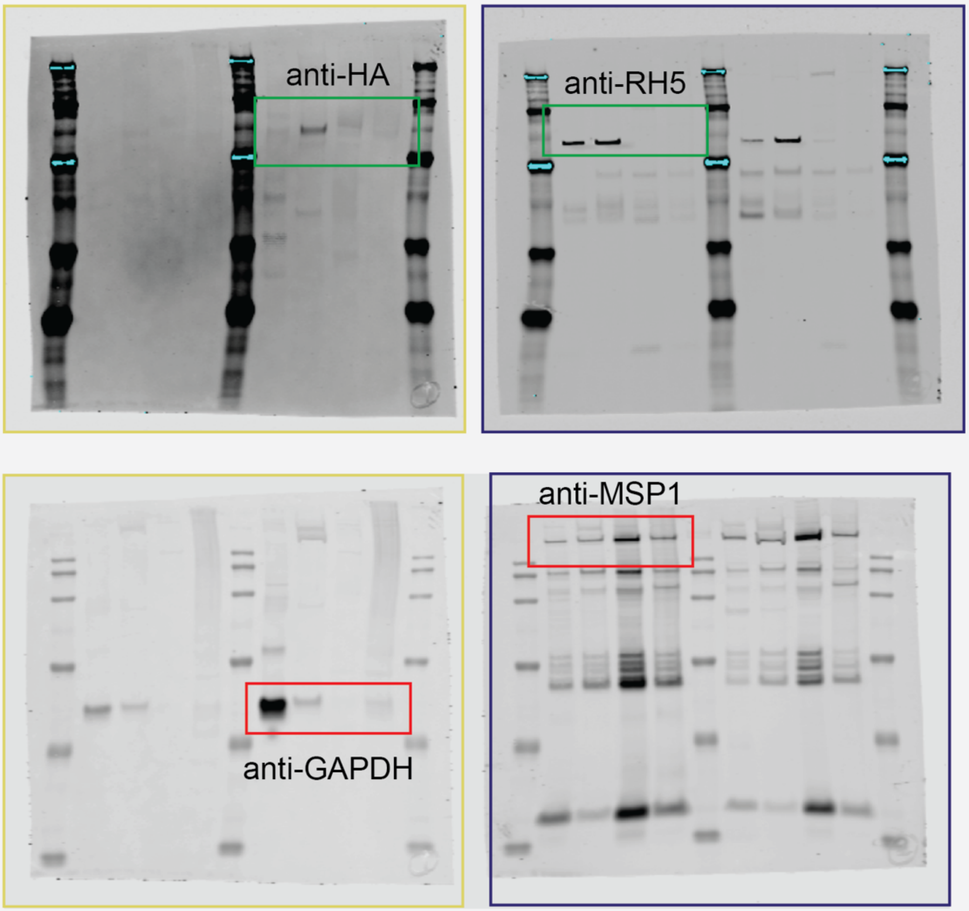

Supplementary figure 19. Full agarose gels and western blots used in Figure 5.

Corresponding figures and antibodies used are annotated. Bands that were cropped in Figure 5 have been boxed according to the colour theme: green (detected in the 800 nm channel), red (detected in the 700 nm channel). Blots probed with different antibody cocktails are boxed according to the colour theme: For Figure 5a blots- yellow (anti-RAP1), purple (anti-HA, anti-GAP45). For Figure 5b blots- yellow (anti-HA, anti-GAPDH), purple (anti-RH5, anti-MSP1-19)

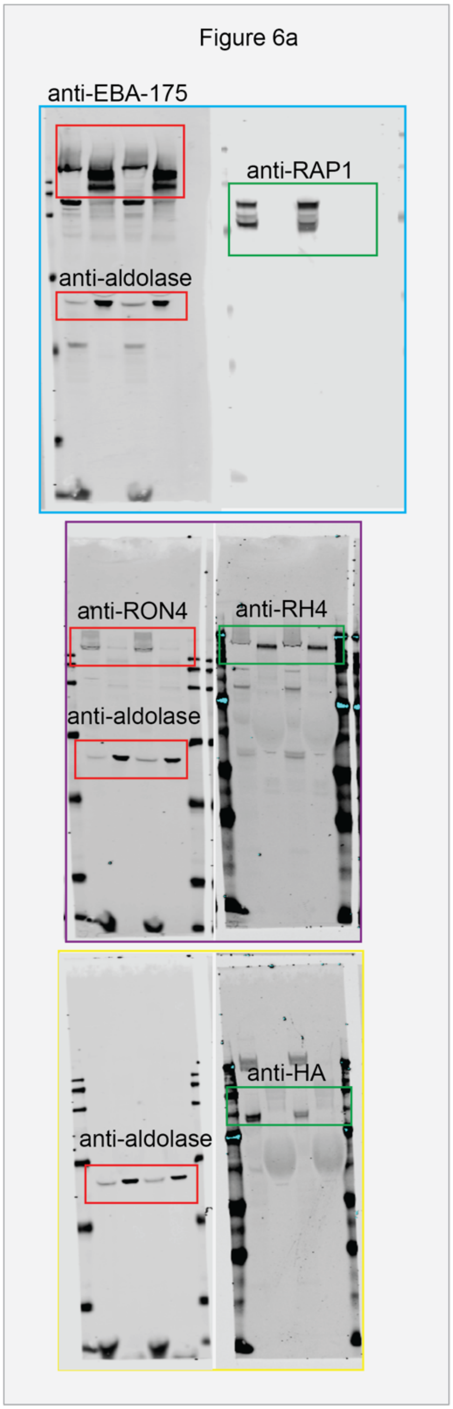
Figure 6a
anti-EBA-175
anti-RAP1
anti-aldolase
anti-RON4
anti-RH4
anti-aldolase
anti-HA
anti-aldolase

Supplementary figure 20. Full agarose gels and western blots used in Figure 6.

Corresponding figures and antibodies used are annotated. Bands that were cropped in Figure 6 have been boxed according to the colour theme: green (detected in the 800 nm channel), red (detected in the 700 nm channel). Membranes were probed with multiple antibodies to detect multiple bands of dissimilar sizes, resulting in multiple banding patterns for some membranes. Blots probed with different antibody cocktails are boxed according to the colour theme: blue (anti-EBA-175, anti-aldolase, anti-RAP1), purple (anti-RH4, anti-RON4, anti-aldolase), yellow (anti-HA, anti-aldolase).

Supp. Figure 9b

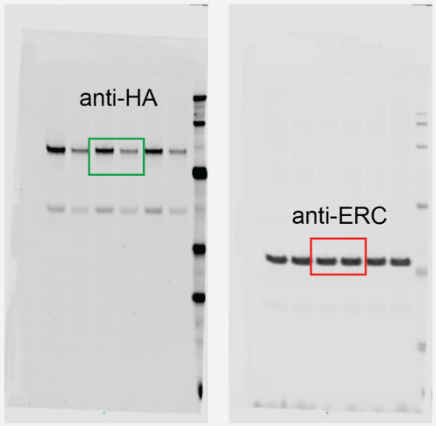

Supp. Figure 10a

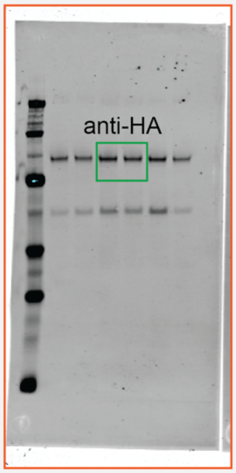

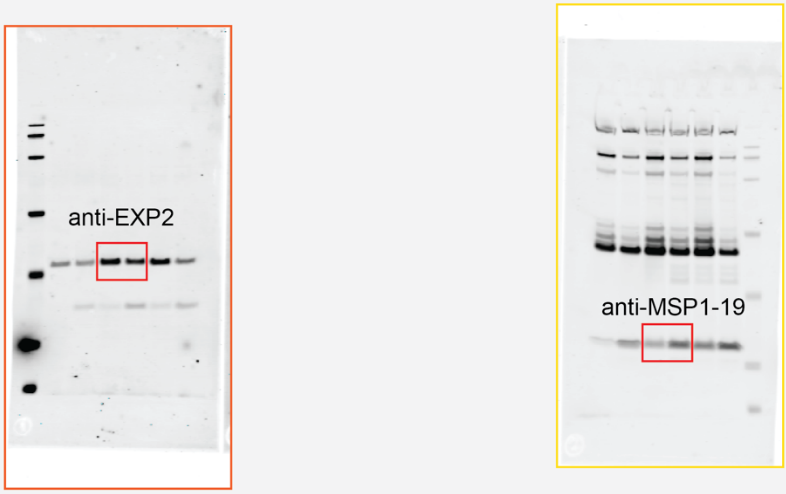

Supp. Figure 11a

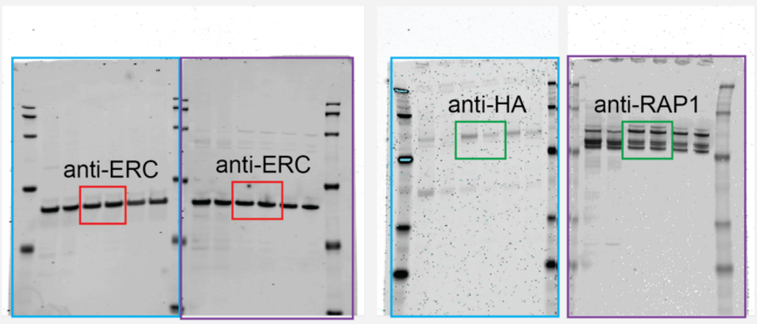

Supplementary figure 21. Full agarose gels and western blots used in Supplementary Figures 9, 10 and 11.

Corresponding figures and antibodies used are annotated. Bands that were cropped in Supplementary Figures 9, 10 and 11 have been boxed according to the colour theme: green (detected in the 800 nm channel), red (detected in the 700 nm channel). Blots probed with different antibody cocktails are boxed according to the colour theme: orange (anti-HA, anti-EXP2), yellow (anti-MSP1-19), blue (anti-HA, anti-ERC), purple (anti-RAP1, anti-ERC).

Figure 15b

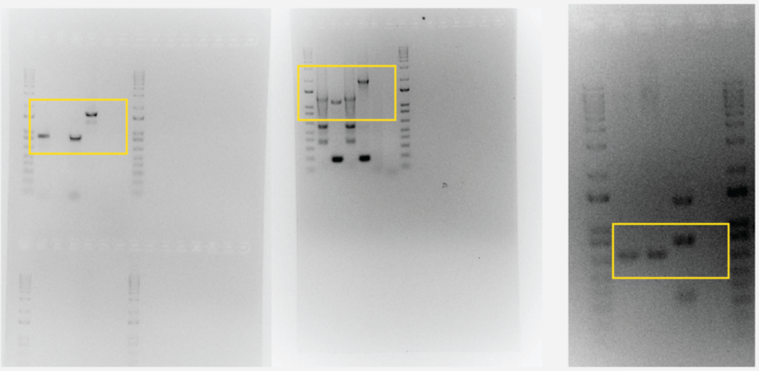

Figure 15c

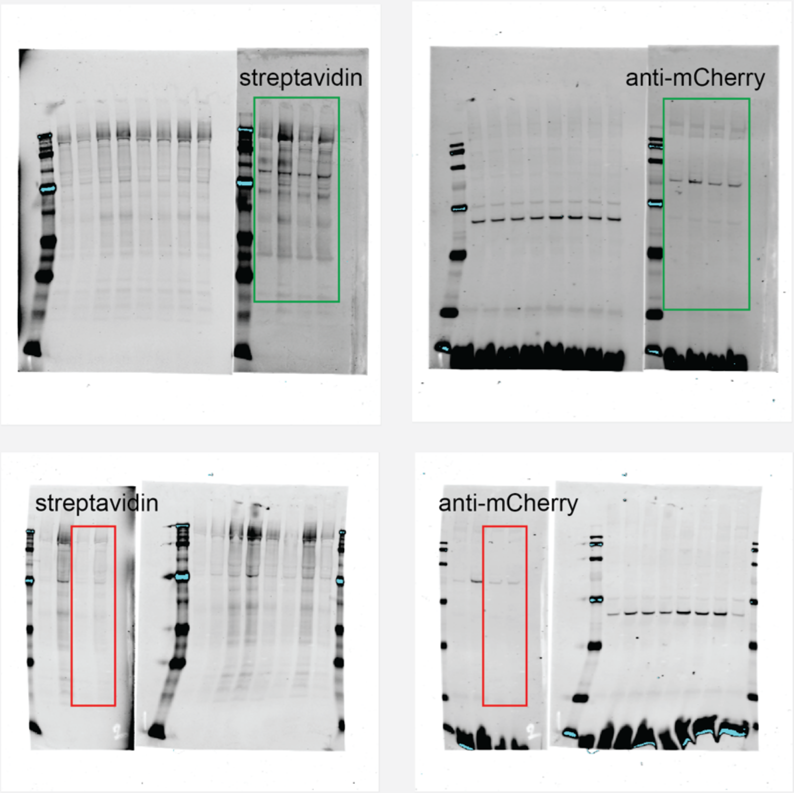

Supplementary figure 22. Full agarose gels and western blots used in Supplementary Figure 15.

Corresponding figures and antibodies used are annotated. Bands that were cropped in Supplementary Figure 15 have been boxed according to the colour theme: yellow (DNA gel), green (detected in the 800 nm channel), red (detected in the 700 nm channel).

SUPPLEMENTARY REFERENCES

1 Liffner, B. *et al.* PfCERLI1 is a conserved rhoptry associated protein essential for *Plasmodium falciparum* merozoite invasion of erythrocytes. *Nature Communications* 11, 1411, doi:10.1038/s41467-020-15127-w (2020).
