# Supplementary material for: Cell biological analysis reveals an essential role for Pfcerli2 in erythrocyte invasion by malaria parasites
Source: Commun Biol. 2022 Feb 9;5:121. doi: 10.1038/s42003-022-03020-9 (PMC8828742; doi:10.1038/s42003-022-03020-9)
Supplement: Supplementary file 3 — Description of Additional Supplementary Files [file 42003_2022_3020_MOESM3_ESM.pdf]

Description of Additional Supplementary Files

File name: Supplementary Data 1
Description: Summary of CERLI2 repeat structures in Laverania.

File name: Supplementary Data 2
Description: Gene structure of cerli1, cerli2 and their homologues in Apicomplexa and Chromerids.

File name: Supplementary Data 3
Description: Average Gene structure of cerli1 and cerli2 lineage genes.

File name: Supplementary Data 4
Description: Summary of PHIS containing proteins in P. falciparum.

File name: Supplementary Data 5
Description: Source Data.
Fig 2e: PfCERLI2 protein levels with knock - down.
Fig 2f: Parasite growth with PfCERLI2 knock -down.
Fig 3a: Parasite invasion with PfCERLI2 knock -down.
Fig 3b: Mean merozoites per schizont with PfCERLI2 knock -down.
Fig 3d: % schizonts with PfCERLI2 knock - down.
Fig 3e: % free merozoites with PfCERLI2 knock -down.
Fig 3g: % bound merozoites with PfCERLI2 knock -down.
Fig 3h: % rings with PfCERLI2 knock -down.
Fig 3j: Merozoite invasion score with PfCERLI2 knoc k -down.
Fig 3k: Diameter of the AMA1 ring with PfCERLI2 knock -down.
Fig 4b: Correlation coefficient with PfCERLI1HA (PCC)
Fig 6b: Invasion ligand protein expression with PfCERLI2 knock -down.
Fig 6 c : RAP1 processing with PfCERLI2 knock -down.
Fig 6e: Rhoptry length with PfCERLI2 knock - down.
Fig 7g: Rhoptry length with PfCERLI2 Page 7 of 19 knock-down using 3D EM.
Fig 7h: Rhoptry surface area with PfCERLI2 knock-down using 3D EM.
Fig 7i: RAP1 distance from nucleus with PfCERLI2 knock-down using 3D EM.
Fig 7j: RON4 distance from nucleus with PfCERLI2 knock-down using 3D EM.
Suppl Fig 14a: RON4 area with PfCERLI2 knock-down.
Suppl Fig 14b: RON4 volume with PfCERLI2 knock-down.
Suppl Fig 14c: RON4 sphericity with PfCERLI2 knock-down.
Suppl Fig 14d: RAP1 area with PfCERLI2 knock-down.
Suppl Fig 14e: RAP1 volume with PfCERLI2 knock-down.
Suppl Fig 14f: RAP1 sphericity with PfCERLI2 knock-down.
