# Supplementary material for: Cell biological analysis reveals an essential role for Pfcerli2 in erythrocyte invasion by malaria parasites
Source: Commun Biol. 2022 Feb 9;5:121. doi: 10.1038/s42003-022-03020-9 (PMC8828742; doi:10.1038/s42003-022-03020-9)
Supplement: Supplementary file 9 — Reporting Summary [file 42003_2022_3020_MOESM9_ESM.pdf]

nature research

Corresponding author(s): Dr Danny Wilson

Last updated by author(s): Nov 24th, 2021

Reporting Summary

Nature Research wishes to improve the reproducibility of the work that we publish. This form provides structure for consistency and transparency in reporting. For further information on Nature Research policies, see our Editorial Policies and the Editorial Policy Checklist.

Statistics

For all statistical analyses, confirm that the following items are present in the figure legend, table legend, main text, or Methods section.

n/a	Confirmed	
☐	☒	The exact sample size (*n*) for each experimental group/condition, given as a discrete number and unit of measurement
☐	☒	A statement on whether measurements were taken from distinct samples or whether the same sample was measured repeatedly
☐	☒	The statistical test(s) used AND whether they are one- or two-sided<br>*Only common tests should be described solely by name; describe more complex techniques in the Methods section.*
☒	☐	A description of all covariates tested
☒	☐	A description of any assumptions or corrections, such as tests of normality and adjustment for multiple comparisons
☐	☒	A full description of the statistical parameters including central tendency (e.g. means) or other basic estimates (e.g. regression coefficient) AND variation (e.g. standard deviation) or associated estimates of uncertainty (e.g. confidence intervals)
☒	☐	For null hypothesis testing, the test statistic (e.g. *F*, *t*, *r*) with confidence intervals, effect sizes, degrees of freedom and *P* value noted<br>*Give P values as exact values whenever suitable.*
☒	☐	For Bayesian analysis, information on the choice of priors and Markov chain Monte Carlo settings
☒	☐	For hierarchical and complex designs, identification of the appropriate level for tests and full reporting of outcomes
☐	☒	Estimates of effect sizes (e.g. Cohen's *d*, Pearson's *r*), indicating how they were calculated

Our web collection on statistics for biologists contains articles on many of the points above.

Software and code

Policy information about availability of computer code

Data collection	Super-resolution microscopy: Zeiss LSM800 with Airyscan<br>Confocal microscopy: Olympus FV3000<br>Western blot imaging: Li-COR Odyssey<br>Flow cytometer: BD Accuri c6<br>Structure prediction: Phyre2 & I-TASSER.<br>Serial block face - scanning electron microscopy: ThermoScientific VolumeScope<br>Array Tomography: FEI Teneo SEM
Data analysis	Microscopy image processing and analysis: Imaris version 9<br>Statistical analysis and graph generation: PRISM version 8<br>Measuring of rhoptry bulb diameter: ImageJ Version 2.0.0<br>Pairwise distance calculation: Geneious version 9.1.3<br>Analysis of flow cytometry data: FlowJo Version 10<br>Quantification of Western blots: ImageStudioLite Version 5.2.5<br>Array Tomography: 3dmod and FIJI<br>Mass spectrometry: Spectronaut

For manuscripts utilizing custom algorithms or software that are central to the research but not yet described in published literature, software must be made available to editors and reviewers. We strongly encourage code deposition in a community repository (e.g. GitHub). See the Nature Research guidelines for submitting code & software for further information.

Data

Policy information about availability of data

All manuscripts must include a data availability statement. This statement should provide the following information, where applicable:

- Accession codes, unique identifiers, or web links for publicly available datasets
- A list of figures that have associated raw data
- A description of any restrictions on data availability

The mass spectrometry proteomics data have been deposited to the ProteomeXchange Consortium via the PRIDE69 partner repository with the dataset identifier PXD028937. All other data available upon request.

Field-specific reporting

Please select the one below that is the best fit for your research. If you are not sure, read the appropriate sections before making your selection.

- [x] Life sciences
- [ ] Behavioural & social sciences
- [ ] Ecological, evolutionary & environmental sciences

For a reference copy of the document with all sections, see nature.com/documents/nr-reporting-summary-flat.pdf

Life sciences study design

All studies must disclose on these points even when the disclosure is negative.

Sample size

Figure 2c: Representative image shown
Figure 2d: Representative image shown
Figure 2e: Representative image shown
Figure 2f: Each datapoint represents 4 biological replicates, with approximately 50,000 cells counted per sample
Figure 3a: Each datapoint represents 4 biological replicates, with approximately 50,000 cells counted per sample
Figure 3b: Each datapoint represents a biological replicate with merozoites countred from 20 parasites counted per replicate
Figure 3c: Each datapoint represents a biological replicate, with approximately 50,000 cells counted per sample
Figure 3d: Each datapoint represents a biological replicate, with approximately 50,000 cells counted per sample
Figure 3e: Representative images shown
Figure 3f: Representative images shown
Figure 3g: Each datapoint represents a biological replicate, with 1000 Red blood cells counted per treatment per replicate
Figure 3h: Each datapoint represents a biological replicate, with 1000 Red blood cells counted per treatment per replicate
Figure 4a: Representative images form 3 biological replicates, with each replicate having 6 images
Figure 4b: Each datapoint represents a single quantified image, from one of 3 biological replicates, with each replicating having 6 images
Figure 5a: Representative blots shown
Figure 5b: Representative blots shown
Figure 6a: Representative blots shown
Figure 6b: Each datapoint represents a quantified western blot
Figure 6c: Each datapoint represents a quantified western blot
Figure 6d: Representative images shown
Figure 6e: Each datapoint represents a rhoptry from a single parasite quantified from one out of 38 images
Figure 7a-f: Representative images from 100 merozoites quantified (per treatment)
Figure 7g-h: Each datapoint represents a single quantified rhoptry, form a total of 100 rhoptries quantified per treatment
Figure 7 i-j: Each datapoint represents distance from a single merozoite out of 31 untreated or 32 2.5 mM GLCN, from a total of 12 schizont images
Figure 8 a-e: Representative images shown. Summary tables of 3 proteomics experiments for PfCERLI1 and PfCERLI2 (full dataset deposited on the PRIDE database as indicated in data availability statement).
Supplementary Figure 9a: Representative images shown
Supplementary Figure 9b: Representative image and summary data from 3 independent experiments
Supplementary Figure 9c: Summary data for 3 independent experiments with a total of 20 invading merozoites represented for each treatment.
Supplementary Figure 10a: Representative blots shown
Supplementary Figure 10b: Each datapoint represents a single quantified western blot
Supplementary Figure 10c: Representative images
Supplementary Figure 11a: Representative blots shown
Supplementary Figure 11b: Each datapoint represents a single quantified western blot
Supplementary Figure 12a-d: Each datapoint represents a single quantified rhoptry, from a total of 100 rhoptries quantified per treatment
Supplementary Figure 13a-b: Each image and graph representative of the 31 (untreated) and 32 (2.5 mM GLCN) merozoites
Supplementary Figure 14a-c: Each column graph represents the data from 779 (untreated) or 1042 (2.5 mM GLCN) RON4 foci
Supplementary Figure 14d-f: Each column graph represents the data from 367 (untreated) or 386 (2.5 mM GLCN) RAP1 foci
Supplementary Figure 14g: Each datapoint represents a single quantified rhoptry diameter as assesed by RAP1 foci for 60 untreated RAP1 foci and 62 2.5 mM GLCN treated foci
Supplementary Figure 15 a-c: Representative blots shown

Supplementary Figure 16 a-e: peptide intensity comparisons between repeat experiments for CERLI1 and CERLI2
Supplementary Figure 17: Representative FACS plots shown
Supplementary Figure 18: Unedited gels/blots for Fig 2
Supplementary Figure 19: Unedited gels/blots for Fig 5
Supplementary Figure 20: Unedited gels/blots for Fig 6
Supplementary Figure 21: Unedited gels/blots for Supp Figs 9, 10, 11
Supplementary Figure 22: Unedited gels/blots for Supp Figure 15

Data exclusions

Data were excluded in Fig 3K (excluded data for fully invaded merozoites across all treatments on the basis that the tight-junction would have closed off). The Full data set is represented in Supp Fig 9c.

Replication

Figure 2c: Three biological replicates
Figure 2d: Three biological replicates
Figure 2e: Three biological replicates
Figure 2f: Four biological replicates
Figure 3a: Four biological replicates
Figure 3b: Three biological replicates
Figure 3c: Three biological replicate
Figure 3d: Four biological replicates
Figure 3e: Three biological replicates
Figure 3f: Five biological replicates
Figure 3g: Five biological replicates
FIgure 3h: Five biological replicates
Figure 4a: Three biological replicates
Figure 4b: Three biological replicates
Figure 5a Three biological replicates
Figure 5b: Three biological replicates
Figure 6a: Six biological replicates
Figure 6b: Six biological replicates
Figure 6c: Six biological replicates
FIgure 6d: Three biological replicates
Figure 6e: Three biological replicates
Figure 7a-h: 100 images across a single biological replicate
Figure 7i-j: Three biological replicates
Figure 8: Three biological replicates
Supplementary Figure 9: Three biological replicates
Supplementary Figure 10: Three biological replicates
Supplementary Figure 11: Three biological replicates
Supplementary Figure 12a-b: 100 images across 3 biological replicates
Supplementary Figure 13a-d: Three biological replicates
Supplementary Figure 14a-g: Three biological replicates
Supplementary Figure 15: Three biological replicates
Supplementary Figure 16: Three biological replicates

Randomization

N/A

Blinding

Figure 3e: Giemsa smears were blinded prior to taking images to be representative images to prevent image acquisition bias
Figure 3f: Giemsa smears were blinded prior to taking images to be representative images to prevent image acquisition bi
Supplementary Figure 14: Image file names were blinded prior to manual measurement of merozoite fluorescence
Figure 3g-h: Giemsa smears were blinded before bound merozoites and ring stage parasites were quantified by microscopy
Supplementary Figure 15g: Image file names were blinded prior to manual measurement of rhoptry width
Figure 3i-k: images of invading merozoites were blinded before scoring and measuring of AMA1 ring diameter.

Reporting for specific materials, systems and methods

We require information from authors about some types of materials, experimental systems and methods used in many studies. Here, indicate whether each material, system or method listed is relevant to your study. If you are not sure if a list item applies to your research, read the appropriate section before selecting a response.

Materials & experimental systems

n/a	Involved in the study
☐	☒ Antibodies
☒	☐ Eukaryotic cell lines
☒	☐ Palaeontology and archaeology
☒	☐ Animals and other organisms
☒	☐ Human research participants
☒	☐ Clinical data
☒	☐ Dual use research of concern

Methods

n/a	Involved in the study
☒	☐ ChIP-seq
☐	☒ Flow cytometry
☒	☐ MRI-based neuroimaging

Antibodies

Antibodies used

Commercial antibodies:
Mouse anti-HA (12CA5), Roche, 11583816001
anti-HA-biotin (3F10), Roche, 12158167001
Rabbit anti-aldolase (ab207494) Abcam
Goat anti-Mouse IgG IRDye 800CW, Li-COR Biosciences, 926-32210
Goat anti-Rabbit IgG IRDye 680RD, Li-COR Biosciences, 926-68071
Alexa Fluor 488 Goat anti-Chicken IgY, Life Technologies, A-11039
Alexa Fluor 594 Goat anti-Mouse IgG H+L, Life Technologies, A-11005
Alexa Fluor 647 Goat anti-Rabbit IgG H+L, Life Technologies, A-21245

Non-commercial antibodies:
Primary antibodies rabbit anti-Rh4, mouse anti-RH5, mouse anti-RAP1 rabbit anti-RON4, rabbit anti-MSP1-19, rabbit anti-EBA175 and rabbit anti-GAP45(R728K) were provided by Prof. Alan Cowman at the Walter and Eliza Hall Institute of Medical Research. Rabbit anti-EXP2 was provided by Dr. Paul Gilson at the Burnet Institute. Rabbit anti-ERC and rabbit anti-GAPDH were provided by Prof. Leann Tilley at Bio21 Institute.

Validation

Antibodies previously validated commercially or by the laboratories listed in peer reviewed publications (available in manuscript).

Flow Cytometry

Plots

Confirm that:

☒ The axis labels state the marker and fluorochrome used (e.g. CD4-FITC).

☒ The axis scales are clearly visible. Include numbers along axes only for bottom left plot of group (a 'group' is an analysis of identical markers).

☒ All plots are contour plots with outliers or pseudocolor plots.

☒ A numerical value for number of cells or percentage (with statistics) is provided.

Methodology

Sample preparation

For growth assays: Cells were washed once in PBS, before staining in PBS + Ethidium bromide for 30 minutes. Cells were then washed twice with PBS before flow cytometry analysis.
For invasion assays: To 50 microlitres of cells, 170 microlitres of PBS + Ethidium bromide was added. Cells were stained for 30 minutes in the dark before flow cytometry analysis.

Instrument

BD Acurri C6

Software

FlowJo Version 10

Cell population abundance

For all flow cytometry experiments, between approximately 50,000 and 80,000 events were captured. Of these, typically >90% fell inside the red blood cell FSC/SSC gate. For growth and invasion assays, trophozoite and ring stage populations typically numbered between 1000 and 10000 events, depending on the parasiteaemia of that treatment. For counting free merozoites by flow cytometry, typically between 2000 and 10000 events were captured, again depending on the parasitaemia of that treatment.

Gating strategy

Red blood cell populations were gated by FSC and SSC. Within this RBC population, newly invaded ring-stage parasites were gated on intermediate FITC (GFP) fluorescence and low PE (Ethidium bromide) fluorescence, late-stage parasites were gated as PE (Ethidium bromide) high events. Free merozoites were gated as FITC (GFP) high and PE (ethidium bromide) high events that occurred outside (lower FSC/SSC) of the red blood cell gate. The Gating strategy has been outlined previously (Wilson et al. AAC, 2013) and is cited in the manuscript and is represented in Supp Figure 17.

☒ Tick this box to confirm that a figure exemplifying the gating strategy is provided in the Supplementary Information.
